# Use of Iodine to Biofortify and Promote Growth and Stress Tolerance in Crops

**DOI:** 10.3389/fpls.2016.01146

**Published:** 2016-08-23

**Authors:** Julia Medrano-Macías, Paola Leija-Martínez, Susana González-Morales, Antonio Juárez-Maldonado, Adalberto Benavides-Mendoza

**Affiliations:** ^1^Departamento de Botánica, Facultad de Ciencias Biológicas, Universidad Autónoma de Nuevo LeónSan Nicolás de los Garza, Mexico; ^2^Laboratorio de Fisiología, Departamento de Horticultura, Universidad Autónoma Agraria Antonio NarroSaltillo, Mexico; ^3^Consejo Nacional de Ciencia y Tecnología, Departamento de Horticultura, Universidad Autónoma Agraria Antonio NarroSaltillo, Mexico; ^4^Departamento de Botánica, Universidad Autónoma Agraria Antonio NarroSaltillo, Mexico

**Keywords:** iodide, iodate, antioxidants, oxidative stress, ROS, nutritional quality

## Abstract

Iodine is not considered essential for land plants; however, in some aquatic plants, iodine plays a critical role in antioxidant metabolism. In humans, iodine is essential for the metabolism of the thyroid and for the development of cognitive abilities, and it is associated with lower risks of developing certain types of cancer. Therefore, great efforts are made to ensure the proper intake of iodine to the population, for example, the iodization of table salt. In the same way, as an alternative, the use of different iodine fertilization techniques to biofortify crops is considered an adequate iodine supply method. Hence, biofortification with iodine is an active area of research, with highly relevant results. The agricultural application of iodine to enhance growth, environmental adaptation, and stress tolerance in plants has not been well explored, although it may lead to the increased use of this element in agricultural practice and thus contribute to the biofortification of crops. This review systematically presents the results published on the application of iodine in agriculture, considering different environmental conditions and farming systems in various species and varying concentrations of the element, its chemical forms, and its application method. Some studies report beneficial effects of iodine, including better growth, and changes in the tolerance to stress and antioxidant capacity, while other studies report that the applications of iodine cause no response or even have adverse effects. We suggested different assumptions that attempt to explain these conflicting results, considering the possible interaction of iodine with other trace elements, as well as the different physicochemical and biogeochemical conditions that give rise to the distinct availability and the volatilization of the element.

This review aims to provide an overview of the biofortification of iodine, presenting the progress in this important area of agricultural research. Information is included about the possible alternative use of iodine as an inductor of abiotic and biotic tolerance. In the literature, a series of reviews focused on human deficiency of iodine resulting from the irregular distribution of the element and its complex and still not well-understood dynamics is available. This review complements the information presented by other authors (Whitehead, 1984; Fuge and Johnson, 1986, 2015; Johnson, 2003; Fuge, 2005, 2013; Steinnes, 2009; Charlton and Skeaff, 2011; Küpper et al., 2011; Moreda-Piñeiro et al., 2011; Pearce et al., 2013) focusing on agronomic efforts and on the comparison of different methods of biofortification applied.

## Iodine dynamics

**Figure 1:**
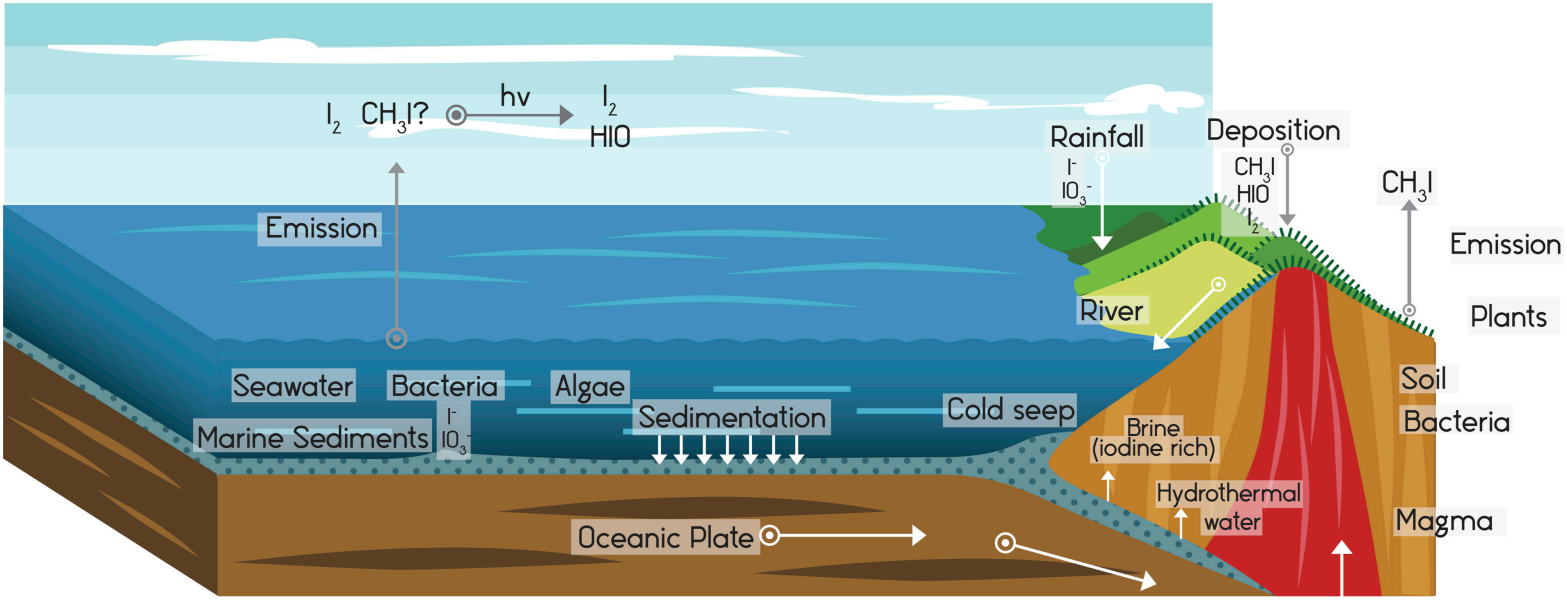
**The global cycle of iodine**. The hydrosphere contains a store of iodine estimated at 7.9 × 10^16^ g. From there it reaches the atmosphere (which stores ~5 × 10^12^ g of iodine) by volatilization (Fuge and Johnson, 1986), forming gases, and aerosols that are brought to land areas and incorporated into terrestrial ecosystems by deposition, precipitation, or absorption. Through new processes of iodine volatilization and dragging on the surface and through underground streams, iodine returns to the ocean. These processes involve significant microbial participation. All compartments of iodine storage are dynamic, being under constant turnover. Adapted from Moreda-Piñeiro et al. (2011).

The oceans are the largest reservoirs of bioavailable iodine on the planet; from there, the element is distributed into the atmosphere and land areas (Fuge, 1996; Venturi, 2011). The second most important reservoir of iodine is the soil, which has a higher content than does its parent material as a result of the activity of the living organisms (Muramatsu and Yoshida, 1999). Approximately 4 × 10^11^ g year^−1^ of iodine volatilize from the ocean into the atmosphere (Miyake and Tsunogai, 1963; Amachi, 2008), with an estimated of 1.14–3.17 × 10^11^ g year^−1^ that volatilizes as CH_3_I (Moore and Groszko, 1999). In the atmosphere, iodine reaches concentrations of 5–20 ng m^−3^ in gaseous forms and 1–5 ng m^−3^ as particulate iodine (Moyers and Duce, 1972). This atmospheric iodine, in the form of I_2_ and organoiodine compounds (CH_3_I and iodinated humic acids), reacts photochemically with O_3_ and forms radicals (I_2_O_2_, I_2_O_3_, and I_2_O_4_) that become transformed into I_2_O_5_. This compound forms particles with a nanometric dimension that induces condensed nuclei for cloud formation (Saunders and Plane, 2005). Iodine in the form of gas and aerosol is carried by the wind and rain to land areas, where it is found in soils mainly in the form of iodide (I^−^) and iodate (${\text{IO}}_{3}^{-}$). In rainwater, the iodine appears at concentration of 2 μg L^−1^ (Bowley, 2013). Once found on land, iodine is distributed in different ways: it is again mobilized by volatilization into the atmosphere by abiotic and biotic processes, fixed in soil and biomass, or dragged to the ocean through water streams (Whitehead, 1984; Moreda-Piñeiro et al., 2011; Saunders et al., 2012; Fuge and Johnson, 2015; Figure 1).

The distribution of iodine between different terrestrial compartments occurs with the significant participation of microbiological processes (Amachi et al., 2003; Amachi, 2008). From a physiological perspective, it is assumed that the flux of iodine among different organisms is valuable as a source of antioxidant potential (Crockford, 2009; Venturi, 2011), as well as by the metabolic value of the compounds resulting from the reaction between the amino acid tyrosine and iodine, such as thyroxine (T4) and its derivatives (T2 and T3; Eales, 1997; Heyland and Moroz, 2005). From an ecological standpoint, the iodine flux between the different layers of the Earth, ecological compartments, and organisms may be considered part of the global system of energy dissipation (Karnani and Annila, 2009).

**Figure 2:**
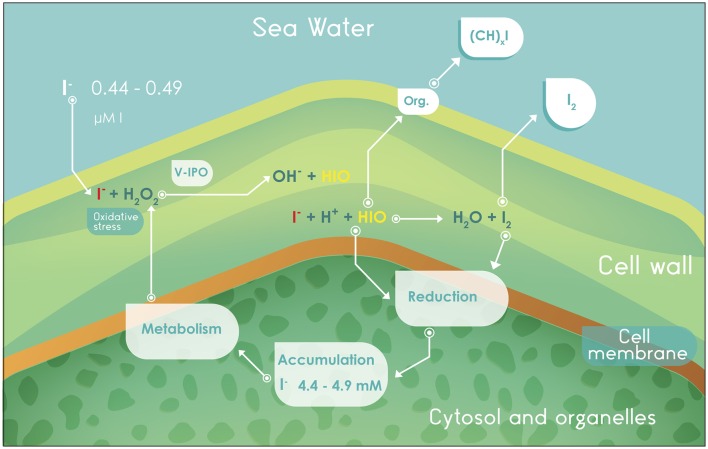
**Absorption, metabolism, accumulation, and volatilization of iodine in algae of the genus ***Laminaria*****. V-IPO, iodoperoxidase enzyme dependent on vanadium; Org., organic compounds.

Among the biological processes of iodine mobilization, one receiving close attention involves iodine metabolism by seaweeds of the genus *Laminaria* (Leblanc et al., 2006), which volatilize iodine through the production of molecular iodine (I_2_) and organoiodine compounds (CH_3_I and CH_2_I_2_; Moore and Groszko, 1999; Carpenter et al., 2000; Leblanc et al., 2006; Jones et al., 2010), coupling the process with the antioxidant metabolism to reduce oxidative stress (Küpper et al., 2008; Nitschke et al., 2013). It has been shown that one iodoperoxidase enzyme dependent on vanadium (V-IPO) is critical in this antioxidant system (Figure 2).

The I^−^ that is absorbed from seawater reacts with H_2_O_2_, (catalyzed by V-IPO) to produce hypoiodous acid (HIO; Küpper et al., 1998). The oxidative process is named peroxide-dependent diffusion (PDD) and occurs in macroalgae, bacteria, and animals outside of the chordates (Miller and Heyland, 2013). The resulting HIO may (i) diffuse into the cytoplasm to be accumulated, (ii) react to form volatile organoiodine compounds, or (iii) react in the apoplast with I^−^ to produce I_2_, which can also migrate to the cytoplasm and be stored or volatilized (McFiggans et al., 2004; Leblanc et al., 2006). Figure 2 partially explains the value of iodine as an antioxidant. Iodide may be a source of reduction potential to the cellular system, once oxidized it can be used for metabolic purposes, stored at an intracellular iodine pool or, to avoid excessive accumulation, dissipated by means of volatile organoiodine compounds or I_2_ sublimation.

It has not been proven that iodine plays a central antioxidant role in land plants as in the *Laminaria* macroalgae, although it has been reported that in the presence of iodine, crop plants increase their antioxidant levels (Gupta et al., 2015). It is assumed that during their evolution, land plants decreased their dependency on iodine as an inorganic antioxidant, developing a whole new series of organic antioxidants (such as ascorbic acid, polyphenols, and carotenoids) in response to the low concentration of available iodine in the emerged areas (Venturi, 2011). Notwithstanding the apparent non-dependency on iodine, higher plants absorb iodine through their roots and leaves, and dissipate it (Barry and Chamberlain, 1963; Whitehead, 1979; Amiro and Johnston, 1989) using halogen methyltransferases not dependent on vanadium (Landini et al., 2012). Similar to iodine, the vanadium is an element of low bioavailability in land areas (Cappuyns and Swennen, 2014); thus it is possible that iodine oxidases have evolved as a dependent on one-carbon metabolism. However, the question of whether vanadium is essential for plants, coupled with the possibility that plant responses to iodine occur under a context of low bioavailability of vanadium has not been fully resolved (Pilbeam and Drihem, 2007). Additionally, marine photosynthetic organisms, algae, and diatoms synthesize analogs to thyroxine (Heyland and Moroz, 2005; Crockford, 2009), and the land plants have transthyretin-like proteins with sequence homology to transthyretin (TTR), the protein for thyroxine transport (Eneqvist et al., 2003; Pessoa et al., 2010); thus, it is possible that iodine may have metabolic functions that are not yet understood in land plants. In animals, the evolutionary strategy to address the low iodine availability in land areas is different: animals are still dependent on iodine as an indispensable element, and iodine is stored in vertebrates in the follicular tissue of the thyroid. In other groups, such as invertebrates and prochordates, iodine is accumulated in other tissues or specific proteins (Eales, 1997). Animal organisms obtain iodine mostly from food intake and, to a lesser extent, through the absorption from drinking water and from gas exchange during breathing (Vought et al., 1970; Whitehead, 1984; Fuge and Johnson, 2015).

## Iodine and human health

**Figure 3:**
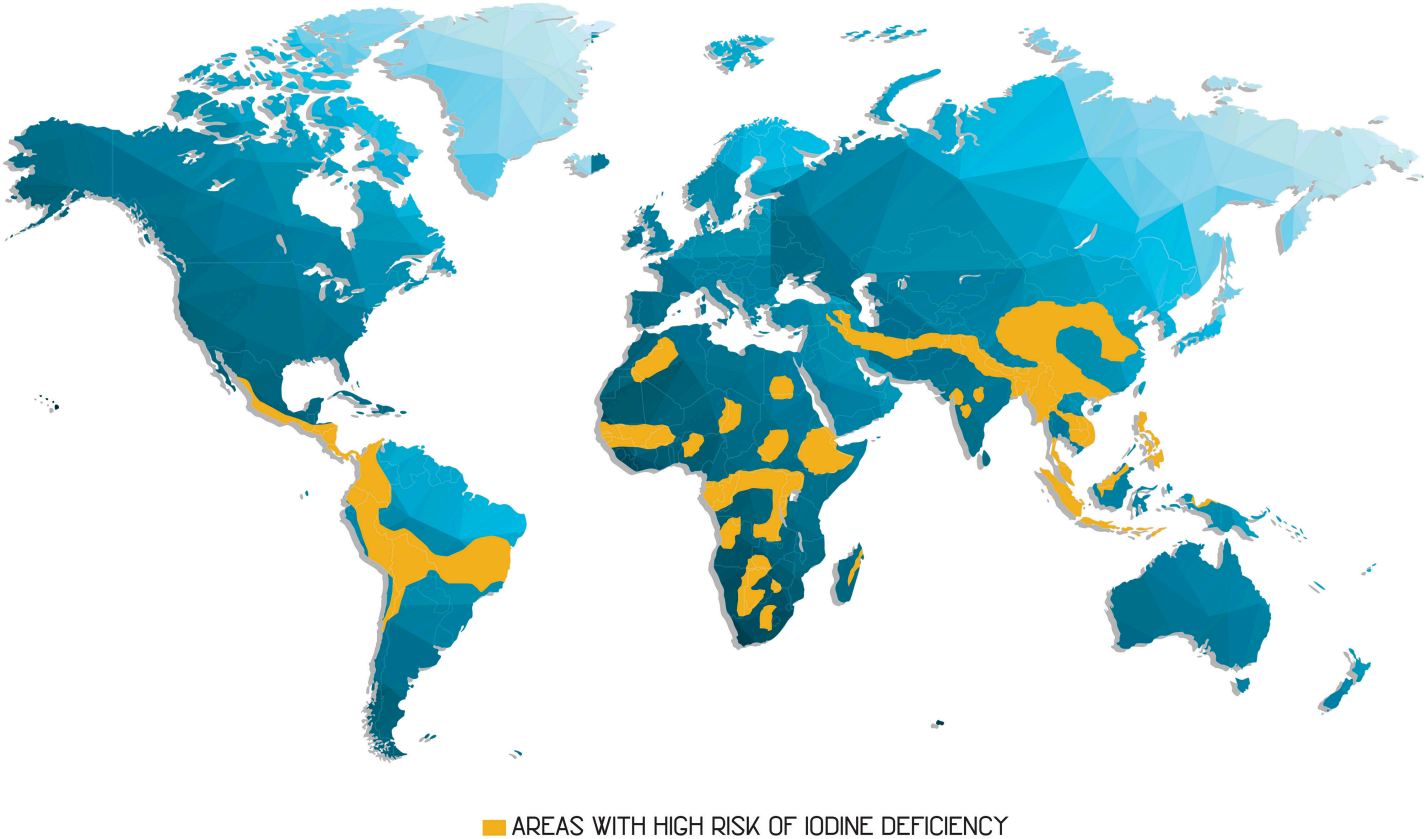
**Regions where the population presents a high risk of iodine deficiency are marked in orange on the map**. Adapted from Burlingame (2013).

According to the World Health Organization (WHO), iodine deficiency (Figure 3) is among the most common nutritional deficiencies, along with those of iron (Fe), zinc (Zn), and vitamin A (Burlingame, 2013; Prasad, 2013).

From the perspective of human health, iodine is one of the most studied elements because of its metabolic importance and because of the complexity associated with the factors that induce its deficiency. Iodine deficiency occurs in many regions of the planet; the irregular distribution of iodine on the Earth's crust is considered as a primal factor (FAO, 2009). An estimated 2 × 10^9^ people ingest an insufficient amount of iodine (Mottiar, 2013), causing the so-called iodine deficiency disorders (IDDs). IDDs refer to illnesses associated with low iodine consumption (Zimmermann et al., 2008).

The best-known IDD due to its prominent symptom is goiter. Nevertheless, the presence of less tangible IDDs has been observed in recent decades, such as an adverse impact on physical and cognitive development in children, and on productivity in adults (Lazarus et al., 2012); it has also been associated with a higher incidence of fetal death, miscarriages, and congenital anomalies (Zimmermann, 2009). On the other hand, it was recently demonstrated that iodine is capable of acting as an antioxidant and as an antiproliferative of malignant cells (Funahashi et al., 2001; García-Solís et al., 2005; Aranda et al., 2013; Anguiano and Aceves, 2011). The daily requirement of iodine, according to the Recommended Dietary Allowances (RDA; World Health Organization, 2007; Andersson et al., 2012), is among 90 and 200 μg day^−1^.

Foods contain different quantities of iodine depending on their origin, individual characteristics, conservation, and preparation. A comprehensive study of the literature (Fordyce, 2003) indicated that the geometric mean iodine concentration in foods is 87 μg kg^−1^, a small amount when considering the daily requirements previously mentioned. Data separation by food type is provided in Table 1.

**Table 1:** **Iodine content in different foods (Fordyce, 2003)**.

**Food**	**I concentration (μg kg^−1^)**
Sea fish	1455.9
Freshwater fish	102.8
Leafy vegetables	88.8
Dairy	83.9
Other vegetables	80.1
Meats	68.4
Cereals	56.0
Fresh fruits	30.6
Water	6.4

Except for the sea fishes, nearly all foods have a low iodine content. The data presented in Table 1 may vary depending on the site where the foods are collected or produced, but clearly show the need to increase the iodine amount in foods. As an example: it has been suggested that lettuce must contain 50–100 μg of iodine per 100 g of fresh weight (Lawson et al., 2015), that is, values several times higher than those shown in Table 1.

Numerous attempts to mitigate the deficit in the consumption of iodine have been made, mainly since the 1920s through the universal iodization of table salt (de Caffarelli, 1997; Zimmermann, 2009; Charlton et al., 2013). However, throughout the years it has been shown that this technique alone is insufficient to ensure the total requirement of iodine (de Benoist et al., 2008), partly because the iodine of table salt is unstable and is subject to many losses by volatilization (Mottiar and Altosaar, 2011). In this regard, the consumption of iodine in organic forms such as seaweed and biofortified foods and yeast, is considered more appropriate (Funahashi et al., 2001; Weng et al., 2008a; Kopeć et al., 2015) because those organic sources are more stable than inorganic ones.

The maximum recommended dietary dose of iodine ranges from 1000 μg day^−1^ (Pennington, 1990) in a daily basis, to 2000 μg day^−1^ by not more than 3 weeks (Backer and Hollowell, 2000). Iodine toxicities are not common under normal conditions (Gupta and Gupta, 1998), and humans appear to have a high tolerance to iodine doses <2000 μg day^−1^ (Bulloch, 2014). Frequently, the toxicity by iodine occurs by genetic or physiological predisposition (Rose et al., 2002) or by the use of iodine-based products as disinfectants (Backer and Hollowell, 2000) and medications (Bulloch, 2014).

Therefore, it is necessary to promote the use of techniques, such as the biofortification of crops, to achieve an adequate iodine intake from foods, either as a complement or as an alternative to the inorganic sources of iodine as the table salt.

## Iodine applications in agricultural crops

There have been numerous studies on the application of iodine in various plant species with the purpose of biofortifying crops. The results reported in the literature are variable according to the applied concentration, chemical form used and adopted production system (Tables 2–**4**). An overview is presented on the absorption, transport and volatilization of iodine in plants, followed by summaries reporting the concentrations and chemical forms of iodine that are used in different production systems.

**Table 2:** **Overall effect of the application of iodine in different crops**.

**Crop**	**Main result**	**Main effect**	**References**
Barley (*Hordeum vulgare* L.)	+	Positive effect on growth.	Borst Pauwels, 1961
Beet (*Beta vulgaris* L.)	+	Positive effect on aboveground biomass.	Borst Pauwels, 1961
Cabbage (*Brassica oleracea* var. *capitata*)	−	Severe diminution of biomass. Higher iodine accumulation.	Mao et al., 2014
Cabbage	±	Chlorosis and necrosis occurs at an application rate of 15 kg ha^−1^. No effects on biomass or quality when applied via foliar spray.	Lawson et al., 2015
Canola (*Brassica napus* L.)	+	Slight positive effect on yield.	Mao et al., 2014
Carrot (*Daucus carota* L.)	+	Increases glucose, fructose, sucrose, total sugars, and soluble solids. No effect on biomass.	Smoleń et al., 2014b
Carrot	−	Plant growth decreases when the iodine concentration exceeds 50 mg (kg soil)^−1^.	Hong et al., 2008
Celery (*Apium graveolens* L.)	+	Iodine applied to soil increases the biomass in leafy vegetables.	Dai et al., 2004b
Ceylon spinach (*Basella alba* L.)	+	Higher iodine accumulation at 40 μg L^−1^. Use of fertigation is recommended.	Ujowundu et al., 2010
Chinese cabbage (*Brassica rapa* ssp. *pekinensis*)	+	Iodine applied to soil increases biomass.	Dai et al., 2004b
Chinese cabbage	+	Biomass increases at low doses. Toxic effect at higher doses.	Weng et al., 2008c
Chinese cabbage	+	When not applied to soil in excess, iodine increases its concentration in edible vegetables.	Weng et al., 2003
Chinese cabbage	−	Plant growth decreases when the iodine concentration exceeds 50 mg (kg soil)^−1^.	Hong et al., 2008
Lettuce (*Lactuca sativa* L.)	+	Higher accumulation of K, Mg, Ca, Mn, and Cd when applying iodine at any dose, form and/or application method.	Smoleń et al., 2011
Lettuce	+	KIO_3_ < 40 × 10^−6^ M increases SOD, APX, GSH, AA, and antioxidant potential. Improves response to salinity.	Leyva et al., 2011
Lettuce	+	Under saline stress iodine increases foliar mass, antioxidant response, and accumulation of phenolic compounds at 20 and 40 μM KIO_3_.	Blasco et al., 2013
Lettuce	±	KI reduces biomass at 40 μM or higher. KIO_3_ has no effect. Higher concentration of antioxidants with KI.	Blasco et al., 2008
Lettuce	+	KI decreases SOD and increases CAT, ascorbate, and glutathione. KIO_3_ increases SOD, APX, CAT, and ascorbate, and has no negative effect on biomass.	Blasco et al., 2011
Lettuce	+	Increases the content of I.	Kopeć et al., 2015
Lettuce	−	Plant growth decreases when the iodine concentration exceeds 50 mg (kg soil)^−1^.	Hong et al., 2008
Lettuce	−	In combination with selenium, iodine has a negative effect on biomass. Negative correlation between I content and K, Mg, Ca, S, Na, B, Cu, Fe, Mn, Zn, Cd, and Pb concentration.	Smoleń et al., 2015b
Lettuce	−	Chlorosis and necrosis occurs at an application rate of 15 kg ha^−1^. No effects on biomass or quality when applied via foliar spray.	Lawson et al., 2015
Lettuce	+	Se + I had no effect on biomass or mineral composition. Synergic interaction between both compounds for absorption through leaves via foliar spray.	Smoleń et al., 2014a
Lettuce	+	No effect on biomass. Iodine concentration in leaves increases with iodine treatment.	Voogt et al., 2010
Linseed (*Linum usitatissimum* L.)	+	Positive effect on growth.	Borst Pauwels, 1961
Maize (*Zea mays* L.)	−	Negative effect on biomass.	Caffagni et al., 2011
Maize	−	Iodine reduces yield.	Mao et al., 2014
Mustard (*Brassica nigra* (L.) W.D.J. Koch)	+	Positive effect on growth.	Borst Pauwels, 1961
Nopal (*Opuntia ficus-indica* (L.) Mill.)	±	Increases ascorbic acid. Decreases fresh and dry weight. Diverse effects on minerals. Changes in some histologic variables.	García-Osuna et al., 2014
Oat (*Avena sativa* L.)	−	Negative effect on growth.	Borst Pauwels, 1961
Onion (*Allium cepa* L.)	+	No effect on the biomass of fruit and root vegetables.	Dai et al., 2004b
Parsley (*Petroselinum crispum* Mill.)	+	Positive effect on growth.	Borst Pauwels, 1961
Potato (*Solanum tuberosum* L.)	−	Reduction of biomass.	Mao et al., 2014
Potato	−	Negative effect on biomass.	Caffagni et al., 2011
Pumpkin (*Cucurbita pepo* L.)	+	Higher iodine accumulation at 40 μg L^−1^. Use of fertigation is recommended.	Ujowundu et al., 2010
Radish (*Raphanus sativus* L.)	+	Concentration of free amino acids increases.	Strzetelski et al., 2010
Radish	±	When not applied to soil in excess, iodine increases its concentration in edible vegetables.	Weng et al., 2003
Rice (*Oryza sativa* L.)	−	KI and KIO_3_ at 100 μM cause biomass reduction.	Mackowiak and Grossl, 1999
Rice	−	KI > 0.25% decreases plant height, panicle length, grain number, and yield.	Singh et al., 2012
Rice	−	Negative effect on growth.	Kato et al., 2013
Ryegrass (*Lolium perenne* (Lam.) Husnot.)	+	Positive effect on the aboveground biomass.	Borst Pauwels, 1961
Soybean (*Glycine max* (L.) Merr.)	+	Increases biomass.	Mao et al., 2014
Soybean	+	SOD, APX, and GR increased with IO_3_^−^.	Gupta et al., 2015
Spinach (*Spinacia oleracea* L.)	+	Iodine application to soil increases biomass.	Dai et al., 2004b
Spinach	+	Slight positive effect on aboveground biomass.	Borst Pauwels, 1961
Spinach	±	When not applied to soil in excess, iodine increases its concentration in edible vegetables.	Weng et al., 2003
Spinach	+	IO_3_^−^ increase iodine concentration in leaves.	Dai et al., 2006
Spinach	−	KI ≥ 10 × 10^−6^ M is toxic. KIO_3_ slightly affects biomass.	Zhu et al., 2003
Spinach	+	Higher absorption through fertigation. No damage to plants.	Smoleń and Sady, 2012
Spinach	+	KIO_3_ increases biomass when applied to nutrient solution. It absorbs more than KI.	Smoleń et al., 2015a
Tobacco (*Nicotiana tabacum* L.)	−	Negative effect on biomass.	Caffagni et al., 2011
Tomato (*Solanum lycopersicum* L.)	+	Extended shelf life.	Limchoowong et al., 2016
Tomato	+	Positive effect on aboveground biomass.	Borst Pauwels, 1961
Tomato	+	KIO_3_ increases soluble solids, fructose, glucose, ascorbic acid, and phenols. Higher iodine accumulation with salicylic acid.	Smoleń et al., 2015c
Tomato	+	Decreases plant weight and accelerated flowering with higher yields.	Lehr et al., 1958
Tomato	−	Plant growth decreases when iodine concentration exceeds 50 mg (kg soil)^−1^.	Hong et al., 2008
Tomato	−	Negative effect on biomass.	Caffagni et al., 2011
Tomato	−	Biomass decreases as the concentration of iodine increases. Ascorbic acid decreases.	Hageman et al., 1942
Tomato	+	Significant increase in iodine without damage to fruits.	Kiferle et al., 2013
Tomato	+	Iodine was taken up when supplied with nutrient solution and leaf spray, and stored in fruits and vegetative tissues.	Landini et al., 2011
Turnip (*Brassica rapa* ssp. *rapa*)	±	Positive effect on aboveground biomass and negative effect on roots.	Borst Pauwels, 1961
Water spinach (*Ipomoea aquatica* Forssk.)	+	Positive effect on growth of low doses of iodine. I− increases vitamin C, while IO_3_^−^ and CH_2_ICOO^−^ decreases it. I^−^ and IO_3_^−^ increases nitrates.	Weng et al., 2008c
White clover (*Trifolium repens* L.)	+	Positive effect on aboveground biomass.	Borst Pauwels, 1961
Wheat (*Triticum aestivum* L.)	−	Negative effect on biomass.	Caffagni et al., 2011
Wheat	+	Positive effect on growth.	Borst Pauwels, 1961
Wheat	−	Decreases biomass.	Mao et al., 2014
Zucchini (*Cucurbita pepo* var. *melopepo*)	+	Higher iodine accumulation at 40 μg L^−1^. Use of fertigation is recommended.	Ujowundu et al., 2010

*Main result: (+), positive; (−), negative; (±), mixed results*.

### Absorption and metabolism of iodine

Iodine is an element that can be absorbed by the root and in aerial structures both by the stomata and by the cuticular waxes with high degree of unsaturation and great capacity to take iodine (Shaw et al., 2007; Tschiersch et al., 2009), both in dissolved form and in gas form as I_2_ and CH_3_I. The impact of the differences among species in the profile and quantity of cuticular waxes on leaf iodine absorption has not been verified. This information may be relevant considering that the cuticular waxes interacting with iodine can be alternatives for the pre- and post-harvest biofortification of fruits and seeds.

There is no information to indicate how much of the element that has been taken up by the plants comes from the soil and how much from the atmosphere, but it is known that the absorption of iodine in gas form can be significant (Barry and Chamberlain, 1963; Nakamura and Ohmomo, 1984; Whitehead, 1984). In opposite Tsukada et al. (2008) estimated the atmospheric contribution to the iodine uptake of rice to be only 0.2%. The direct atmospheric contribution would be expected to be higher in regions near the sea and lower in continental areas; however, the evidence indicates that the volatilization of iodine that is fixed in the soil may also be an important factor in iodine transfer to organisms (Whitehead, 1984; Fuge and Johnson, 2015).

Once the iodine is absorbed, it is transported through the xylem, finding that its redistribution through the phloem is low (Herrett et al., 1962); thus it accumulates in greater amounts in leaves than in fruits and seeds. However, in lettuce plants treated with iodine by leaf spray Smoleń et al. (2014a) found evidence of iodine transport from leaves to the roots. In wheat plants, even when iodine was applied by foliar spraying, the mobility from the leaves to the grains (termed the translocation factor) was very low (0.2–1.1%), but this value appears to be cumulative, i.e., iodine moves from the leaves to the grain with each application event (Hurtevent et al., 2013). The observed translocation factors for radish, potato, and bean range from 0.8 to 2.6%, 0.1 to 2.3%, and 0.1 to 2.6%, respectively (Henner et al., 2013). On the other hand, the iodine transfer factor (ITF) refers to the element that is absorbed by the root, and is defined as the ratio of the iodine concentration in the plant tissues to its concentration in the substrate. ITF is higher in leafy crops such as spinach (ITF ≥ 2.0), than in fruits such as tomatoes and nectarines, or cereal grains (0.0005 ≤ ITF ≤ 0.02; Shinonaga et al., 2001; Lawson, 2014). For example, starting with a soil concentration of 48 mg kg^−1^, the distribution of the iodine that is absorbed by a rice plant (dry weight) is as follows: 53 mg kg^−1^ in the root, 16 mg kg^−1^ in the leaves, and 0.034 mg kg^−1^ in the polished grain (Tsukada et al., 2008).

When iodine is applied to plants as ${\text{IO}}_{3}^{-}$ it is reduced to I^−^ by the action of an iodate reductase, which responds to the availability of iodine in the medium (Kato et al., 2013). This reductase activity also occurs in microorganisms (Amachi, 2008), but the magnitude of the microbial contribution in the soil process is unknown. In soils ${\text{IO}}_{3}^{-}$ is more efficient taken up by plants compared to I^−^ (Lawson et al., 2015), and in soilless cultures the application of I^−^ induces toxicity more easily in plants than does ${\text{IO}}_{3}^{-}$ (Borst Pauwels, 1962; Umaly and Poel, 1971; Muramatsu et al., 1983; Zhu et al., 2003). The lower toxicity of ${\text{IO}}_{3}^{-}$ could be explained by the iodate as an alternative substrate to other abundant enzymes, such as nitrate reductase (Barber and Notton, 1990), or by the activation of iodate reductase through ${\text{IO}}_{3}^{-}$ inducing other responses associated with redox signaling and iodine metabolism in plants, in addition to the reduction of ${\text{IO}}_{3}^{-}$.

**Figure 4:**
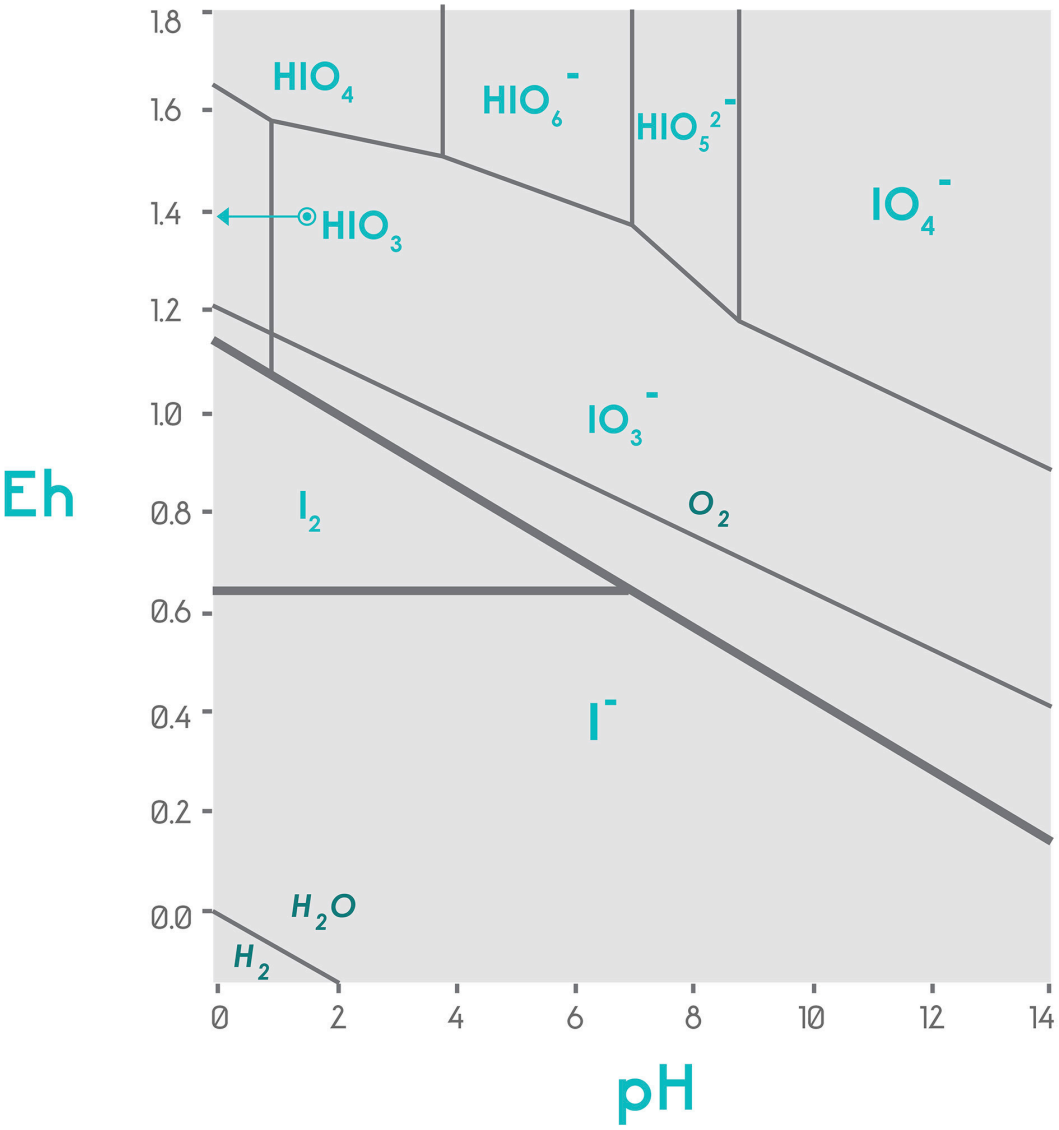
**Stability diagram of the chemical species iodide and iodate concerning the potential of hydrogen (pH) and oxidoreductive potential (Eh) in volts**. The graph is centered on thermodynamic considerations and displays the stability of the different iodine species in the Eh-pH plot. Under conditions of low redox potential (Eh ≤ 0.65), independently of pH, iodine is available in the form of iodide; under low pH and 0.65 < Eh < 0.85, I_2_ will be the predominant form. When Eh > 1.0, the oxidized forms of iodine are observed, with ${\text{IO}}_{3}^{-}$ being the most abundant in a wide pH range. Adapted from Vinogradov and Lapp (1971).

Since ${\text{IO}}_{3}^{-}$ is more thermodynamically stable than I^−^, it is hypothesized that it is the most likely form to be available in agricultural soils. However, because the I^−^/${\text{IO}}_{3}^{-}$ ratio depends on biological activity, it is not limited strictly to a thermodynamic balance (Kaplan et al., 2014), as shown in Figure 4. This fact makes it difficult to predict the pattern of iodine speciation in a particular soil.

Plants absorb iodine as I^−^ through ionic channels and chloride transporters that are energized by proton pumps (White and Broadley, 2009); therefore, there may occur interference scenarios with other anions such as nitrate, thiocyanate, and perchlorate (Voogt and Jackson, 2010). The identity of the I^−^ transporters is not firmly established, but their activity can presumably be shared by several families of transporters and anion channels (White and Broadley, 2009; Landini et al., 2012). Among these are the Na:K/Cl cotransporters belonging to the *CCC* family of genes (Colmenero-Flores et al., 2007), which directly regulate the concentration of ions in the root xylem (Shabala, 2013; Wegner, 2014; Fricke, 2015). Another group is the gene family of CLC Cl^−^-channels permeable to I^−^ (Roberts, 2006; Barbier-Brygoo et al., 2011). Currently, the *CLC* genes have been linked to osmotic stress tolerance (Ma et al., 2016; Nguyen et al., 2016), stomatal movement, nutrient transport, and heavy metal tolerance (Zifarelli and Pusch, 2010).

Once absorbed, transported, and accumulated in different plant organs, iodine is not stable; plants volatilize iodine as methyl iodide (CH_3_I) using the enzymes halide ion methyltransferase (HMT) and halide/thiol methyltransferase (HTMT), with methyl transferase activity dependent on S-adenosylmethionine (SAM; Redeker et al., 2004; Itoh et al., 2009). The affinity of these methyl-halide transferase enzymes to iodine is much greater than that observed in other halogens or ions such as thiocyanate (Takekawa and Nakamura, 2012). Volatilization can be faster as iodine increases its concentration in the substrate (Itoh et al., 2009), and occurs in all organs; in rice, the iodine foliar concentration decreases exponentially with a half-life of 14 days (Nakamura et al., 1986). Therefore, the action of methyltransferases continuously reduces the iodine store present in plants (Landini et al., 2012). It has been noted that iodine is a phytotoxic element *per se* to the plants and that volatilization is a detoxification mechanism (Saini et al., 1995) or a by-product of the methyltransferase activity reactions that occur in plants (Redeker et al., 2004). An alternative explanation is that the iodine is toxic only depending on the environmental context: for example, under conditions of high solubilization or little fixation, as previously mentioned (Yuita, 1994); when a deficiency of some elements such as Fe or Cu (perhaps vanadium) occurs; or merely as a result of the intrinsic differences between plant species to metabolize iodine (Saini et al., 1995).

There is little information about the differences between vegetable species and the environmental factors that affect the activity of enzymes that dissipate iodine, but it is known that the activity increases with temperature and changes in different developmental phases, peaking during the reproductive stage (Muramatsu and Yoshida, 1995; Redeker et al., 2004). This activity is also positively associated with the iodine absorption rate of the nutrient solution and the irradiance; in addition, the stomata partially regulate the flow of iodine from the mesophyll into the atmosphere (Amiro and Johnston, 1989). It has been reported that in bacteria, the iodine volatilization activity increases under stress (Li et al., 2012, 2014). This metabolic activity of iodine volatilization by plants contributes to the overall activity of volatilization that occurs in soils and inland waters and is part of the global flow of iodine. In soils, both I^−^ as ${\text{IO}}_{3}^{-}$ are volatilized as HI, HIO_3_, and HIO_4_ (Sheppard et al., 1994). In soil-plant systems, the emission of iodine into the atmosphere occurs both by abiotic soil components and by plants and soil microorganisms (Ban-nai et al., 2006), but the total activity increases with the presence of vegetation (Muramatsu and Yoshida, 1995). The estimated amount of iodine that is volatilized for soil-plant system range from 0.28 to 0.62 kg ha^−1^ season^−1^, with an average of 0.40 kg ha^−1^ season^−1^ (Redeker et al., 2002). If we consider this value alongside the global average iodine content in soil (2.6 mg kg^−1^; Watts et al., 2010), and consider that topsoil represents 1.5 × 10^6^ kg ha^−1^, then without exogenous iodine inputs the volatilization activity will exhaust the soil iodine reservoir in 9 years.

The success of the biofortification of crops depends on more than the iodine application technique (Sheppard et al., 1994; Fuge and Johnson, 2015). Iodine is a dynamic element that is under constant turnover by living organisms, including plants and humans (Küpper et al., 2011), as well as by the ecological systems components (Kaplan et al., 2014). The ideal effort would pay direct attention not only to plants but also to the entire system of which they are a part. Iodine is metabolized by the complete ecological system, possibly under an overall control scheme that is not yet well understood, and it is advisable to consider other biotic and abiotic factors as part of the biofortification strategy. For example, the role of the rhizosphere as a complex and dynamical environment where interacts the roots, microorganisms, and inorganic soil components have been little explored regarding the absorption of iodine (Terzano et al., 2015), but has proven to be an useful approach in the case of Fe and Si (Pii et al., 2015; Gattullo et al., 2016). In the same way, there is a notable absence of studies on the biofortification of cultivated fungi or the use of mycorrhizae or plant growth-promoting rhizobacteria to modify the absorption of iodine, although this approach has proven its value for other elements, such as Fe and Zn (He and Nara, 2007; Pellegrino and Bedini, 2014; Rana et al., 2015). Mycorrhizae further decrease the toxicity of certain elements to plants (Leung et al., 2013), an issue that has not been reported for iodine.

An additional point is that other factors, climatic, ecological, phytochemical, and cultural (e.g., food preparation and storage), may decrease the bioavailability or stability of iodine and avoid the correlation between iodine distribution and its presence in the human population (Stewart et al., 2003; Kotwal et al., 2007; Longvah et al., 2012; Zia et al., 2015).

### Speciation and complexation of iodine in soil

The iodine content of soil is the result of the complex dynamic balance of three processes: incorporation from the atmosphere, fixation, and volatilization, resulting in an enormous range of variation of iodine contents in the soil, from <0.1 to 150 mg (kg soil)^−1^ (Moreda-Piñeiro et al., 2011). The interaction of iodine with soil organic and inorganic components increases the fixation, decreases the rate of volatilization, and reduces its bioavailability. The complexation of iodine with organic matter, metal oxides and clays causes a strong fixation of iodine in soil and modifies the concentration of water-soluble iodine available to plants (Whitehead, 1974). The soluble iodine content of soils is usually <10% of the total iodine fixed in soil. The availability of soluble iodine is higher with a low oxidoreductive potential (Eh) (with I^−^ as the dominant chemical form of iodine) and lower under oxidizing conditions (with ${\text{IO}}_{3}^{-}$ as the most abundant form; Fuge and Johnson, 2015).

The Eh of soil changes the iodine speciation, which is expressed as the dynamic ratio [${\text{IO}}_{3}^{-}$]/[I^−^] (Figure 4). In the soil, organic matter, flooding, excessive irrigation and inorganic sources of reductor potential, such as the sulfides, Fe^+2^, and FeS induce low Eh, for which the expected form of iodine would be I^−^, generated from the reaction ${\text{IO}}_{3}^{-}$+ 6e^−^ + 6H^+^ → I^−^ + H_2_O^1^. This reaction can be developed abiotically with ${\text{IO}}_{3}^{-}$ and organic matter producing HIO and I_2_, the latter of which is reduced to I- (Steinberg et al., 2008). By contrast, under high Eh (e.g., low amount of organic matter or low water content in the soil pores), ${\text{IO}}_{3}^{-}$will be the most abundant form. The difference between ${\text{IO}}_{3}^{-}$ and I^−^ is that the former is less subjected to volatilization (Guido-Garcia et al., 2015).

It has been observed in experiments on different types of soil that ${\text{IO}}_{3}^{-}$ tends to be more adsorbed to soil components than I^−^, also showing less leaching and toxicity toward crops (Hong et al., 2012; Lawson et al., 2015). In soils with >10% organic matter, it has been observed that the humic substances can fix the I^−^ and ${\text{IO}}_{3}^{-}$ from aerosols and rain, whereas in soil with <6% organic matter, the ${\text{IO}}_{3}^{-}$ is adsorbed to metal oxides [Fe(OH)_3_, Al(OH)_3_, Mn_4_O_2_]. With a low pH, or with the Eh to the reducing side, these same metals act as reducers and accelerate the I^−^ reaction with organic matter (Bowley, 2013). In acidic soils (pH < 7), the balance between chemical species of iodine leans toward I^−^; while under pH > 7, the predominant form is ${\text{IO}}_{3}^{-}$. The ITF values for cereal grains do not change in the pH interval 5.4–7.6 (Shinonaga et al., 2001), however, toward the extremes of pH (pH < 5 and pH > 8), more adsorption of I^−^ and ${\text{IO}}_{3}^{-}$ will occur (Yoshida et al., 1992; Dai et al., 2009), probably decreasing the bioavailability of iodine for plants.

Among the different physicochemical factors that modify the availability and immobilization of iodine in the soil, the amount of organic matter is the most widely studied (Whitehead, 1978; Sheppard and Thibault, 1992; Yu et al., 1996; Dai et al., 2006). Humified soil matter has been identified as an important reservoir that decreases the iodine dissipation rate (Shetaya et al., 2012) through the formation of covalent bonds between carbon atoms and iodine (Stavber et al., 2008). This reaction mechanism of iodine with organic matter is an electrophilic substitution of H by iodine in a phenolic ring (Reiller et al., 2006). Soil microbes can accelerate this process through the action of laccase enzymes that oxidizes I^−^ to I_2_ and HOI (Shimamoto et al., 2011; Seki et al., 2013), which in a subsequent step are incorporated into organic matter.

The association of iodine and organic matter is not permanent; it is known that iodine in soil is subjected to absorption and desorption processes. Under reducing conditions, such as those in rice paddy fields, soil suffers high iodine desorption promoted by low Eh (−0.1 V), the addition of straw or glucose, root exudates, and microbial metabolism. The desorbed iodine is volatilized by organic matter (especially with a low level of humification), either directly by abiotic halogenation or by the intervention of haloperoxidases of microorganisms (Wever, 2012; Leri and Ravel, 2015). The initial reaction with organic matter is faster for I^−^ and slower for ${\text{IO}}_{3}^{-}$, but after a time of ~60 days or more, both forms participate in a process of transformation in volatile organoiodine compounds, occurring in the presence of organic matter in the soil, with or without the participation of microorganisms (Yamaguchi et al., 2010). Volatilization is dependent on both Eh and pH, with the lowest levels of volatilization in oxic alkaline soils, where the most abundant form of iodine is the non-volatile ${\text{IO}}_{3}^{-}$; in contrast, in waterlogged and organic soils occur higher rates of volatilization resulting from the predominance of I^−^ that is oxidized to I_2_ and CH_3_I. It has been suggested that in inland areas, the contribution of atmospheric iodine depends significantly on the volatilization of the iodine in the soil (Fuge and Johnson, 2015).

### Impact on productivity and yield

The use of iodine in the nutrient solution in concentrations from 10^−6^ M (equivalent to 0.13 mg L^−1^) in soilless culture typically produces an increase in biomass in leafy vegetables, such as Chinese cabbage, spinach, and lettuce (Borst Pauwels, 1961; Whitehead, 1973; Weng et al., 2003, 2008c; Zhu et al., 2003; Dai et al., 2004b; Blasco et al., 2013). The iodine concentration can be increased to 10^−5^ M (equivalent to 1.3 mg L^−1^), to produce biofortified leafy vegetables using I^−^, ${\text{IO}}_{3}^{-}$, or iodoacetic acid (CH_2_ICOOH^−^; Weng et al., 2008c). While Lehr et al. (1958) obtained higher yields of tomato when applying 2 mg L^−1^ KI, Hageman et al. (1942) could not observe any effect on the biomass of tomato when applying 3.2 × 10^−5^ M KI (equivalent to 4 mg L^−1^). Meanwhile, in tomato, Kiferle et al. (2013) used high concentrations of 1 to 5 × 10^−3^ M of KI and 0.5 to 2 × 10^−3^ M KIO_3_ in the nutrient solution once a week (eight times, starting with the fruit set of the first cluster), obtaining remarkable results, with an accumulation of iodine in the fruit of up to 10 mg of iodine per kg of fresh weight of fruit with little phytotoxicity. In strawberry plants, iodine increases plant biomass and fruit quality (Li et al., 2016). On the other hand, a decrease in biomass has been reported in tomato and potato (Caffagni et al., 2011), as well as in carrot (Smoleń et al., 2014b) and in *Opuntia* (García-Osuna et al., 2014), although in other plants where the vegetative reserve organs are also harvested, such as onion, iodine seems to have no effect on the weight of the plant (Dai et al., 2004b). Moreover, in rice, decreases in weight (Mackowiak and Grossl, 1999) and plant height (Singh et al., 2012) occur when applying potassium iodide. This negative effect does not seem to be general for grasses, considering that null or positive effects on biomass have been reported in wheat and corn (Borst Pauwels, 1961; Mao et al., 2014). Furthermore, when iodine is applied to the soil where plants are growing, the results are either mixed, showing positive, null, and negative effects (Dai et al., 2004b), or, as in tomato, are less efficient in terms of iodine bioaccumulation in the fruits compared to the soilless system (Caffagni et al., 2012).

It has also been observed that the effect of the application of iodine on biomass was directly dependent on the amount applied. Globally, the average concentration of iodine in the soil is 2.6 mg kg^−1^ (Watts et al., 2010). Contributions of up to 10 mg (kg soil)^−1^ promote plant growth, whereas values greater than 50 mg (kg soil)^−1^, which are used to increase the iodine concentration significantly in plant tissues, produce varying results depending on the plant species (Cui et al., 2003; Lawson, 2014). In some species, such as Chinese cabbage, the application of more than 25 mg (kg soil)^−1^ decreases the plant biomass (Hong et al., 2008). On the other hand, iodine concentrations higher than 100 μM in the nutrient solution revealed an adverse effects on rice biomass (Mackowiak and Grossl, 1999; Singh et al., 2012), and in lettuce the same negative effect was observed by adding 40 μM of iodine (Blasco et al., 2008; Table 2). In strawberry plants, iodine in nutrient solution at concentrations of up to 1.97 × 10^−6^ M (0.25 mg L^−1^) of I^−^ and 2.86 × 10^−6^ M (0.50 mg L^−1^) of ${\text{IO}}_{3}^{-}$ increased the plant biomass and iodine concentration in fruits (Li et al., 2016). Thresholds for beneficial concentrations and toxicity of iodine are different between species, as a result of the inherent variability found among species and of the specific interaction of each plant species with edaphic, climatic, and biotic variables (Hageman et al., 1942; Mackowiak et al., 2005; Caffagni et al., 2012).

The results in the literature indicate that neither the chemical species applied nor the form of iodine application (i.e., fertigation, foliar spray, nutrient solution) has a consistent effect between different crops (Tables 3, 4). It has been reported that the application of iodine as ${\text{IO}}_{3}^{-}$ is favorable to that of I^−^, especially for the synthesis of antioxidant compounds (Leyva et al., 2011; Blasco et al., 2013), although in some species such as strawberry, clover, and perennial ryegrass, I^−^ is more efficient than ${\text{IO}}_{3}^{-}$ (Whitehead, 1973; Li et al., 2016). In lettuce, KIO_3_ applied to the soil at up to 7.5 kg ha^−1^ IO_3_ is more effective than KI, as it gave a better result in terms of biofortification (50–100 μg I per 100 g FW) and does not affect biomass negatively. In contrast, by leaf spraying the best result was obtained when applying KI at 0.5 kg ha^−1^ iodide (Lawson et al., 2015). The above indicates that each species will respond in a different way and under the context of the culture system. The difference between ${\text{IO}}_{3}^{-}$ and I^−^ becomes more complicated in the case of cultivation in soil due to the different stability, residuality, and form of interaction of each chemical species with biotic and abiotic soil components (Dai et al., 2004a, 2009). A possible alternative for the biofortification of crops grown in soil is the use of marine algae applied to soil (Fuge and Johnson, 2015), or mixtures of marine algae and diatomite, with the algae being the source of iodine and diatomite an adsorbent that provides a constant supply of the element. The mixture achieves a good result in terms of the biofortification of different crop species (Weng et al., 2013, 2014). A second alternative would be the application of iodine in plantlets, using enriched peat, perhaps iodine complexed with biopolymers or porous materials, or leaf spray. This biofortification technique in the pre-transplanting stage has worked well in case of cucumber biofortified with selenium (Businelli et al., 2015).

**Table 3:** **Overall effect of the application of iodine according to the applied chemical form**.

**Chemical form**	**Main result**	**Main effect**	**Author(s)**
CH_2_ICOONa	−	Decreased vitamin C. Higher absorption than that of I^−^ and IO_3_^−^.	Weng et al., 2008c
Kelp algae based fertilizer	+	Iodine applied to soil increases its concentration in edible vegetables.	Weng et al., 2003
Iodated organic fertilizer	+	Iodine absorption increased with the application of iodated organic fertilizer.	Weng et al., 2013
NaI	±	Increases biomass at low doses. Toxic at high doses.	Weng et al., 2008a
NaI	±	At 0.033 × 10^−6^ M concentration biomass increases, while at 0.66 × 10^−6^ M biomass decreases.	Weng et al., 2008b
NaIO_3_	+	Increases biomass.	Weng et al., 2008b
NaIO_3_	±	Increases biomass at low doses. Toxic at high doses, but less toxic than NaI and KI.	Weng et al., 2008a
NaIO_3_	−	Decreases vitamin C and increases nitrates.	Weng et al., 2008c
KI	±	Increases biomass at low doses. Toxic at high doses.	Weng et al., 2008a
KI	−	Plant growth decreases when iodine concentration exceeds 50 mg (kg soil)^−1^.	Hong et al., 2008
KI	−	Chlorosis and necrosis occurs at an application rate of 15 kg ha^−1^. No effects on biomass or quality when applied via foliar spray.	Lawson et al., 2015
KI	+	No effect on biomass. Iodine concentration in leaves increases with iodine treatments.	Voogt et al., 2010
KI	+	Higher absorption through fertigation. No damage to plants.	Smoleń and Sady, 2012
KI	−	Absorption more difficult than that of KIO_3_.	Smoleń et al., 2015a
KI	−	KI induces less growth than does KIO_3_	Borst Pauwels, 1961
KI	−	KI reduces biomass at 40 μM or higher. Higher concentration of antioxidants.	Blasco et al., 2008
KI	+	Decreases SOD, and increases CAT, ascorbate, and glutathione.	Blasco et al., 2011
KI	+	Higher accumulation of K, Mg, Ca, Mn, and Cd when applying iodine in any dose, form and/or application method.	Smoleń et al., 2011
KI	+	Increases I content.	Kopeć et al., 2015
KI	+	Greater accumulation than KIO_3_.	Smoleń et al., 2015b
KI	±	Increases ascorbic acid. Decreases fresh and dry weight. Increases Cu and Mn.	García-Osuna et al., 2014
KI	±	Increases glucose, fructose, sucrose, total sugars, and soluble solids, and decreases biomass. Higher effect than KIO_3_.	Smoleń et al., 2014b
KIO_3_	+	KIO_3_ < 40 × 10^−6^ M increases SOD, APX, GSH, ascorbic acid, and antioxidant potential. Improves response to salinity.	Leyva et al., 2011
KIO_3_	+	Increases SOD, APX, CAT, and ascorbate, with no negative effect on biomass.	Blasco et al., 2011
KIO_3_	±	Biomass decreases at all concentrations, but KIO_3_ has a less negative effect than that of KI.	Caffagni et al., 2011
KIO_3_	±	No damage or biomass diminution at concentrations of 1 and 10 μM, while 100 μM has a slightly negative effect.	Mackowiak and Grossl, 1999
KIO_3_	−	Slight negative effect on biomass and growth.	Zhu et al., 2003
KIO_3_	+	No effect on biomass. Iodine accumulation is independent of dose.	Voogt et al., 2010
KIO_3_		Higher absorption through fertigation. No damage to plants.	Smoleń and Sady, 2012
KIO_3_	+	Biofortification without damage to fruits.	Kiferle et al., 2013
KIO_3_	±	Chlorosis and necrosis occurs at an application rate of 15 kg ha^−1^. No effects on biomass or quality when applied via foliar spray. More efficient to biofortification than KI.	Lawson et al., 2015
KIO_3_	+	KIO_3_ increases biomass when applied to nutritive solution. Iodine is better absorbed with KIO_3_ than using KI.	Smoleń et al., 2015a
KIO_3_	+	KIO_3_ promotes growth more than does KI.	Borst Pauwels, 1961
KIO_3_	+	No effect on biomass. Increased phenols and ascorbic acid at 80 μM and increased antioxidant potential at 120 μM.	Blasco et al., 2008
KIO_3_	+	Higher iodine accumulation at 40 μg L^−1^. Adding iodine to soil promotes plant absorption.	Ujowundu et al., 2010
KIO_3_	+	Under saline stress iodine increases foliar mass, antioxidant response and the accumulation of phenolic compounds at 20 and 40 μM KIO_3_.	Blasco et al., 2013
KIO_3_	+	IO_3_^−^ is recommended as a beneficial compound to cadmium stress.	Gupta et al., 2015
KIO_3_	+	KIO_3_ increases soluble solids, fructose, glucose, ascorbic acid, and phenols. Higher iodine accumulation in combination with salicylic acid.	Smoleń et al., 2015b
KIO_3_	+	Increases ascorbic acid. Decreases fresh and dry weight. Increased P, K, Mg, and Fe. Changes in xylem and mucilage.	García-Osuna et al., 2014
KIO_3_	+	Increases glucose, fructose, sucrose, total sugars, and soluble solids and decreases biomass. Less effect than KI.	Smoleń et al., 2014b
KIO_3_	+	Se + I has no effect on biomass or mineral composition. Synergic interaction between both compounds for absorption through leaves via foliar spray.	Smoleń et al., 2014a

*Main result: (+), positive; (−), negative; (±), mixed results*.

**Table 4:** **Overall effect of iodine application according to the application method**.

**Via**	**Main result**	**Main effect**	**Author(s)**
Fertigation	+	Significant increase in iodine accumulation without damage to fruits.	Kiferle et al., 2013
Fertigation	+	Higher iodine accumulation in leaves.	Smoleń et al., 2015a
Fertigation	+	Iodine applied to soil increases the biomass in leafy vegetables.	Dai et al., 2004b
Fertigation	+	Higher iodine accumulation at 40 μg L^−1^. Use of fertigation is recommended.	Ujowundu et al., 2010
Fertigation	+	Higher absorption through fertigation. No damage to plants.	Smoleń and Sady, 2012
Fruit	+	Extended shelf life.	Limchoowong et al., 2016
Leaf spray	−	Plants accumulate less iodine through foliar application than in nutrient solution.	Landini et al., 2011
Leaf spray	+	Concentration of free amino acids increases in radish.	Strzetelski et al., 2010
Leaf spray	+	Higher accumulation of K, Mg, Ca, Mn, and Cd when applying iodine at any dose, form, and/or application method.	Smoleń et al., 2011
Leaf spray	+	Se + I has no effect on biomass or mineral composition. Synergic interaction between both compounds for absorption through leaves via foliar spray.	Smoleń et al., 2014a
Leaf spray	+	No effect on biomass or quality. Higher iodine accumulation in lettuce than when applied to soil.	Lawson et al., 2015
Nutrient solution	+	Decreases plant weight and accelerated flowering with higher yields.	Lehr et al., 1958
Nutrient solution	+	KIO_3_ promotes growth more than KI at early development stages.	Borst Pauwels, 1961
Nutrient solution	−	KI at concentrations of 10 and 100 μM causes biomass reduction.	Mackowiak and Grossl, 1999
Nutrient solution	−	KI causes severe damage. KIO_3_ has a slight effect on biomass. Higher iodine concentration in nutrient solution improves iodine accumulation in plants.	Zhu et al., 2003
Nutrient solution	+	Increasing levels from 0 to 1 mg L^−1^ linearly increases the absorption of iodine in the three chemical species. A linear correlation between the iodine content in the roots and in the aboveground part is observed.	Weng et al., 2008c
Nutrient solution	+	Biomass increases at low doses of iodine. Toxic effect at higher doses.	Weng et al., 2008a
Nutrient solution	+	Plants accumulate more iodine when applied in nutrient solution than through foliar spray.	Landini et al., 2011
Nutrient solution	+	KIO_3_ < 40 × 10^−6^ M increases SOD, APX, GSH, ascorbic acid, and antioxidant potential. Improves response to salinity.	Leyva et al., 2011
Nutrient solution	+	KIO_3_ increases SOD, APX, CAT, and ascorbic acid. Not phytotoxic.	Blasco et al., 2011
Nutrient solution	+	Increases foliar mass, antioxidant response, and phenolic compounds at 20 and 40 μM.	Blasco et al., 2013
Nutrient solution	±	Increases ascorbic acid. Decreases fresh and dry weight. Diverse effects on minerals. Changes in some histologic variables.	García-Osuna et al., 2014
Nutrient solution	+	In combination with salicylic acid the iodine content in fruits increases. KIO_3_ increases soluble solids, fructose, glucose, ascorbic acid, and phenols.	Smoleń et al., 2015c
Nutrient solution	−	Biomass decreases at all concentrations used.	Caffagni et al., 2011
Nutrient solution	−	Biomass decreases as the concentration of iodine increases. Ascorbic acid decreases.	Hageman et al., 1942
Soil	−	Plant growth decreases when the iodine concentration exceeds 50 mg kg soil)^−1^.	Hong et al., 2008
Soil	−	Chlorosis and necrosis occurs at an application rate of 15 kg ha^−1^.	Lawson et al., 2015
Soil	±	Biomass increases at low doses. Toxic effect at higher doses.	Weng et al., 2008b
Soil	±	Higher accumulation of nitrates in leaves. No damage to plants.	Smoleń and Sady, 2012
Soil	+	High efficiency in biofortification when iodine is applied mixed with humic and fulvic acids.	Smoleń et al., 2015a
Soil	±	When not applied to soil in excess, iodine increases its concentration in edible vegetables.	Weng et al., 2003
Soil	+	Concentration of free amino acids increases.	Strzetelski et al., 2010
Soil	+	Iodine absorption increased with the application of iodated organic fertilizer.	Weng et al., 2013
Soil	+	Iodine application to soil increased iodine accumulation in cabbage leaves.	Mao et al., 2014
Soil	+	SOD, APX, and GR increases with IO_3_.IO_3_ is recommended as a beneficial compound to treat cadmium stress.	Gupta et al., 2015
Soil	+	Increases glucose, fructose, sucrose, total sugars, and soluble solids. No effect on biomass.	Smoleń et al., 2014b

*Main result: (+), positive; (−), negative; (±), mixed results*.

**Figure 5:**
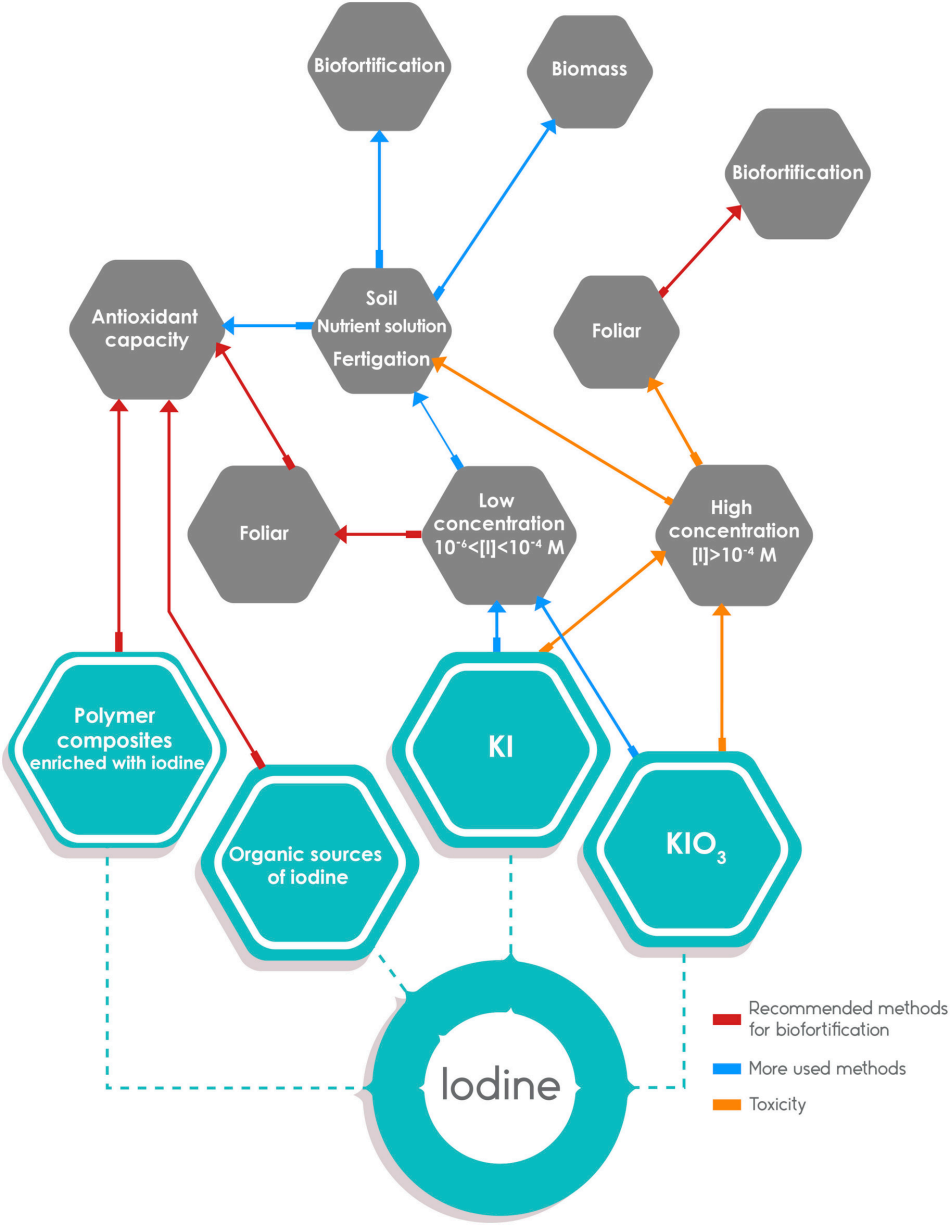
**Graphic summary on the results of different methods of application of iodine**. On the left side, the methods recommended based on the information presented in the review (indicated with red directional arrows): it includes organic sources of iodine applied to the soil and foliar spray with inorganic iodine, and the use of iodine fixed in polymers as a potentially useful method. On the right side, the most widely used application methods with the addition of KI and KIO_3_ to soil or nutrient solution. In this case, the concentration used determines the outcome. The blue arrows indicate a low concentration of iodine with adequate results on biofortification and plant growth. The orange arrows indicate a high concentration of iodine that may lead to biofortification but accompanied by plant toxicity.

Considering the form of iodine application (Table 4), biofortification has been successful with the application of 5% KIO_3_ solution, dripped into irrigation canals (Cao et al., 1994; Ren et al., 2008). Other authors suggest the use of fertigation as a means of iodine application because their results show that adding iodine to the soil increases its absorption by plants (Ujowundu et al., 2010; Kiferle et al., 2013) especially if it is applied together with humic substances or organic acids (Smoleń et al., 2015a,c). The application of iodine through fertigation has shown other positive effects, such as increased biomass in leafy vegetables (Dai et al., 2004b). Foliar spray is another effective method of biofortification of plants with iodine; in lettuce (Smoleń et al., 2014a) and alfalfa (Altınok et al., 2003) this method was more efficient than the application of iodine in the nutrient solution (Figure 5).

### Antioxidant content

Iodine applications have shown mixed effects on the antioxidant potential in several crop species, depending on the sources of iodine, concentration, and type of application. In a study of soybean grown in containers with soil and compost, it was found that the KIO_3_ at concentrations of 20, 40, and 80 μM increased the enzyme activities of SOD and APX (Gupta et al., 2015). In tomato, it was reported that applying ${\text{IO}}_{3}^{-}$ at 7.88 μM increased the content of ascorbic acid and total phenolic compounds (Smoleń et al., 2015c). Similarly, an increase was reported in the content of ascorbic acid in *Opuntia ficus-indica*, grown in soil under low tunnels by applying 10^−4^ M KIO_3_ and KI by fertigation (García-Osuna et al., 2014). In *Ipomoea aquatica*, I^−^ induced a higher amount of ascorbic acid, while ${\text{IO}}_{3}^{-}$ and iodoacetic acid (CH_2_ICOO^−^) had the opposite effect (Weng et al., 2008c). Whereas, in tomato grown in sand, I^−^ at a concentration of 4 mg L^−1^ (3.2 × 10^−5^ M) decreased the concentration of ascorbic acid in the foliage of plants (Hageman et al., 1942).

Blasco and collaborators have extensively studied the impact of iodine on antioxidant metabolism in lettuce grown in hydroponics. In their first study, it was found that the application of KI increases the accumulation of phenols and ascorbic acid, as well as the antioxidant potential (Blasco et al., 2008). Subsequently they reported that the application of KI (20, 40, and 80 μM) and KIO_3_ (20 μM) increased the concentration of ascorbic acid and the enzymatic activity of catalase (CAT) but decreased the GSH concentration and activity of SOD. The APX activity was increased more effectively by KIO_3_ than by KI (Blasco et al., 2011). The positive effect occurred even when applying low concentration (<40 μM) of KIO_3_, increasing the activity of enzymatic antioxidants, such as superoxide dismutase (SOD) and ascorbate peroxidase (APX), as well as that of non-enzymatic antioxidants such as glutathione (GSH) and ascorbic acid (AA) (Leyva et al., 2011). In a more recent study, they found an increase in the antioxidant response and a greater accumulation of total phenolic compounds using KIO_3_ at concentrations of 20 and 40 μM (Blasco et al., 2013).

As shown in Table 3, most of the cited studies used as a source of iodine KI or KIO_3_. There are more reports of negative effects when applying KI, while there are more reports of positive effects when applying KIO_3_, especially in the generation of antioxidant compounds. The difference in effects between the chemical forms of iodine is possible related to the function of ${\text{IO}}_{3}^{-}$ as growth promoter by inducing reductase activity in the root (Kato et al., 2013), while the iodide reduction ability perhaps modifies the redox balance and cell-associated methyltransferase metabolism, making more likely metabolic adjustments that slow the growth and yield. Unfortunately, there is not much research on the effect of iodine on the metabolic processes of plants. In lettuce, the application of iodide and iodate (20, 40, and 80 μM) altered N metabolism and photorespiration. Positive effects were observed on biomass and N uptake with the use of iodate, whereas iodide decreased plant biomass and N concentration (Blasco et al., 2010). In marine plants, iodide does not cause these effects because it is rapidly oxidized by the V-IPO enzyme (Küpper et al., 1998), which has not been found active in land plants (Pilbeam and Drihem, 2007).

On the other hand, the possibility that many of the effects that have been observed when applying iodine modify the behavior and profile of the plant microbiome should not be ignored. Bacteria and fungi dissipate halogens with a metabolic system that includes a V-IPO enzyme (Wever, 2012; Fournier et al., 2014). It is possible that the response of the plant microbiome and, indirectly, the responses of plants to iodine application depend on the characteristics of the soil and irrigation water, including the concentrations of vanadium, sulfur, and organic matter. The importance of the microbiome as a possible determinant of plant responses to iodine has been poorly explored, but its significance has become evident regarding the adaptability and susceptibility of plants to stress (Porras-Alfaro and Bayman, 2011; Berg et al., 2014).

### Stress tolerance

Most of the factors that induce stress increase the concentration of reactive oxygen species (ROS) at the cellular level. Consequently, the induction of antioxidants is considered an important facet of the adaptive responses leading to stress tolerance in plants (Gill and Tuteja, 2010). It has been proposed that iodine was one of the first (inorganic) antioxidants, that allowed the organisms to resist oxidative stress once the atmospheric O_2_ concentration began to increase after the origin of oxygenic photosynthesis (Crockford, 2009; Küpper et al., 2011; Venturi, 2011). This function of iodine was proven in marine algae, where the element inactivates superoxide (${\text{O}}_{2}^{-}$), hydroxyl (OH^.^), singlet oxygen (^1^O_2_), and hydrogen peroxide (H_2_O_2_) (Küpper et al., 2008). In some studies, it was found that iodine increased the amount of antioxidants and allowed greater resistance to certain types of abiotic stress, such as salinity and heavy metals (Leyva et al., 2011; Gupta et al., 2015). The treatment of soybean and sunflower seeds with a dry dressing of iodine and calcium carbonate reduced physiological deterioration under high temperature and humidity. Treated seeds exhibited lower levels of membrane damage, reflected in better germination and seedling growth [Dey(née Pathak) and Mukherjee, 1984]. The short-term pre-treatment of oilseed rape seeds with iodine considerably improved the survival of individuals in deterioration tests (Powell et al., 2005). Seed deterioration is primarily associated with oxidative stress (Sun and Leopold, 1995); therefore the above studies demonstrate the induction of tolerance by iodine, functioning perhaps as an antioxidant. Analogous results were obtained by pretreating peanut seeds with zinc, leading to the improvement in the response to the pathogen *Aspergillus niger* (Jajda and Thakkar, 2012).

More research is needed on the potential of iodine to induce tolerance to stress. The adoption of the use of iodine by the commercial agricultural sector will be fastest if the application of the element is presented to producers and companies as an alternative to mitigate damage from biotic and abiotic stresses and to promote plant growth. The advantage of this approach is that it is more attractive from an economic standpoint, reaching in parallel the goal of crop biofortification. To our knowledge, there are no published studies on the impact of iodine on plant pathogens, although it was reported that iodine is effective (0.7 mg L^−1^) in killing fungi in recirculating water in soilless cultures (Runia, 1995). The problem with the direct use of iodine as a microbicide is that it rapidly induces resistance and forces the use of higher concentrations than are suitable for plants and beneficial microorganisms (Mackowiak et al., 2005). Instead we considered the possibility that iodine is an inductor of tolerance to certain pathogens by activating or by modifying the plant's defense systems through redox signals or through chemical changes in the cuticle (Shaw et al., 2007), which is essential for the induction of systemic acquired resistance (SAR; Xia et al., 2009). The same modifications on cuticular waxes induced by iodine may change the pattern of interactions of plants with pathogens, such as germination and the formation of appressoria (Gniwotta et al., 2005; Silva-Moreno et al., 2016). If it is shown that iodine has this function, the element could be applied as a tool to control crop pathogens.

### Interactions with other elements

The conversion of iodine in its different chemical species, the mobilization and metabolism, depends on the factors that modify Eh and pH. Iodine *per se* has a significant impact on the redox state of the system that absorbs the element (Venturi, 2011); therefore, it interacts with other chemical components of the system, such as organic compounds and metal ions (Fe, Cu, Mn, V), modifying the oxidation state and bioavailability (Hageman et al., 1942). These components in turn give rise to changes in the chemical form, bioavailability and rate of volatilization of iodine in the biomass, water, and soil (Whitehead, 1984), as well as possible changes in the bioavailability of other elements (Terzano et al., 2015).

An iodine biofortification program must occur ideally in absence of restrictions on other mineral elements (White and Broadley, 2009). The interactions between mineral elements can be either synergistic or antagonistic. Synergisms refer to the increased absorption, transport, uptake or metabolism of an element in the presence of iodine. Antagonisms occur when any of the listed activities is diminished in the presence of iodine. In lettuce, it was found that the soil application of KI (0.5–2.0 kg ha^−1^) and leaf spraying with KIO_3_ (0.02–2 kg ha^−1^) do not substantially change the mineral composition of lettuce. From a statistical point of view, there occurred significant changes in plants in both macronutrients N, P, K, Mg, Ca, S, and Na, and microelements B, Cu, Fe, Mn, Zn, and Mo, including Al, Cd, and Pb, but the changes were not significant from a functional point of view (Smoleń et al., 2011). It has been verified in hydroponics that the combined application of KIO_3_ and ${\text{SeO}}_{4}^{-2}$ in lettuce plants does not affect the biomass or mineral composition, showing a synergistic effect resulting in the increased absorption of both elements in the leaves (Smoleń et al., 2014a). The above does not seem to cause any significant antagonism between iodine and other elements, at least for lettuce. However, in a more recent study of the same crop (Smoleń et al., 2015b), a negative correlation was found between the contents of iodine and of K, Mg, Ca, S, Na, B, Cu, Fe, Mn, Zn, Cd, and Pb. Moreover, an increase in P, K, Mg, and Fe was reported when applying KIO_3_, along with an increase in Cu and Mn when using KI in *Opuntia ficus-indica* (García-Osuna et al., 2014). Unfortunately for other species there is little information about the impact of iodine on other mineral elements (Smoleń et al., 2014a).

In biofortification experiments with tomato, where iodine was applied in a range of 10^−6^–10^−5^ M, a positive correlation was found between iodine concentration and Cu and Mn in leaves (Hageman et al., 1942). In lettuce, applying KI to the soil and KIO_3_ by leaf spray produced the same effect on Mn but not on Cu (Smoleń et al., 2011).

Bacteria living in iodine-rich groundwater have proteins called IoxA, which are capable of oxidizing I^−^ to I_2_. These proteins have been characterized as multicopper oxidases, i.e., oxidases with several Cu cofactors (Suzuki et al., 2012; Shiroyama et al., 2015). Another possible explanation regarding the relationship between copper and iodine has to do with the ability of copper oxidases to oxidize I^−^ to I_2_ or HOI (Xu, 1996). It is possible that a greater amount of iodine present in plant tissues induces increased activity in the systems that dissipate the element, such as copper oxidases and possibly other oxidases with Fe and Mn (Klebanoff, 1982; Schlorke et al., 2016). This increased activity could induce changes in the concentration of Cu, Fe, and Mn in tissues that actively dissipate iodine.

Hageman et al. (1942) proposed that changes in the mineral composition of plants that occur when applying iodine relate to a redox phenomenon, explaining that the oxidation of I^−^ to I_2_ provides a reducing potential of −0.535 V. Iodate may cause a similar effect by the induction of reductase activity in the root (Kato et al., 2013). This redox effect of iodine will have a more or less impact on the bioavailability of other elements depending on the complexity and number of components in the interaction. There are fewer of these components in soilless production systems but much more in a soil system with interacting biological, organic and inorganic components in both the exchange matrix and soil solution (Jones, 1998). It is possible that these differences in the complexity of interactions between the components of each production system (soil and soilless) could help to explain the diversity of results obtained with iodine application in crops. The study of ionome in plants might be used to deal with this complexity, as a tool for explain and predict the nutritional profile, considering both essential as non-essential elements. This approach has been successful in the case of iron (Pii et al., 2015; Gattullo et al., 2016), but so far has not been used with iodine.

## Conclusions

The dynamic behavior of iodine is modified by multiple environmental factors that change its flow rate and mobilization among different compartments of the ecosystems. It is necessary to consider more closely the microbial contribution to these flows and to the functions of iodine in ecological systems, in addition to their mere presence or potential role in crop plants.

The variability of the effects of iodine, when applied to crops, can be partially explained when considering the possible impact on N metabolism, photorespiration, and one-carbon metabolism. Additionally, it is necessary to consider interactions with other elements, such as Fe, Mn, Cu, and V, either directly in the plant metabolism or indirectly through the microbiome of the plant.

In general, the effect of iodine is positive on the growth of plants. Good results are obtained regarding biofortification when applied to the soil as KIO_3_ in concentrations of 7.5 kg ha^−1^, 10 mg (kg soil)^−1^ in pots, or 10^−6^–10^−5^ M in the nutrient solution. Leaf spray with KI at 0.5 kg ha^−1^ gave good results. With higher concentrations, the response is variable: negative, neutral, or positive, depending on the plant species.

Positive results have been obtained when applying 10^−3^ M iodine in the nutrient solution but only when doing so on a weekly basis. The use of seaweed applied to the soil also increased the availability of iodine. More research is needed on the use of biopolymers to form complex with iodine to enhance bioavailability and decrease volatilization in soil and pots and on the potential of iodine to induce a higher stress tolerance.

## Author contributions

All authors were responsible for processing information and manuscript writing. AB was responsible for manuscript drafting. All authors read and approved the final manuscript.

### Conflict of interest statement

The authors declare that the research was conducted in the absence of any commercial or financial relationships that could be construed as a potential conflict of interest.

## Footnotes

^1^The reverse reaction is less favorable and less likely in soils.

## References

[B1] Altınok S. Sozudogru-Ok S. Halilova H. (2003). Effect of iodine treatments on forage yields of alfalfa. Commun. Soil Sci. Plant Anal. 34, 55–64. 10.1081/CSS-120017415

[B2] Amachi S. (2008). Microbial contribution to global iodine cycling: volatilization, accumulation, reduction, oxidation, and sorption of iodine. Microbes Environ. 23, 269–276. 10.1264/jsme2.ME08548 21558718

[B3] Amachi S. Kasahara M. Hanada S. Kamagata Y. Shinoyama H. Fujii T.. (2003). Microbial participation in iodine volatilization from soils. Environ. Sci. Technol. 37, 3885–3890. 10.1021/es0210751 12967109

[B4] Amiro B. D. Johnston F. L. (1989). Volatilization of iodine from vegetation. Atmos. Environ. 23, 533–538. 10.1016/0004-6981(89)90002-4

[B5] Andersson M. Karumbunathan V. Zimmermann M. B. (2012). Global iodine status in 2011 and trends over the past decade. J. Nutr. 142, 744–750. 10.3945/jn.111.149393 22378324

[B6] Anguiano B. Aceves C. (2011). Iodine in mammary and prostate pathologies. Curr. Chem. Biol. 5, 177–182. 10.2174/187231311796765049

[B7] Aranda N. Sosa S. Delgado G. Aceves C. Anguiano B. (2013). Uptake and antitumoral effects of iodine and 6-iodolactone in differentiated and undifferentiated human prostate cancer cell lines. Prostate 73, 31–41. 10.1002/pros.22536 22576883

[B8] Backer H. Hollowell J. (2000). Use of iodine for water disinfection: iodine toxicity and maximum recommended dose. Environ. Health Perspect. 108, 679–684. 10.1289/ehp.00108679 10964787 PMC1638306

[B9] Ban-nai T. Muramatsu Y. Amachi S. (2006). Rate of iodine volatilization and accumulation by filamentous fungi through laboratory cultures. Chemosphere 65, 2216–2222. 10.1016/j.chemosphere.2006.05.047 16828143

[B10] Barber M. J. Notton B. A. (1990). Spinach nitrate reductase : effects of ionic strength and ph on the full and partial enzyme activities. Plant Physiol. 93, 537–540. 10.1104/pp.93.2.537 16667499 PMC1062546

[B11] Barbier-Brygoo H. De Angeli A. Filleur S. Frachisse J.-M. Gambale F. Thomine S.. (2011). Anion channels/transporters in plants: from molecular bases to regulatory networks. Annu. Rev. Plant Biol. 62, 25–51. 10.1146/annurev-arplant-042110-103741 21275645

[B12] Barry P. J. Chamberlain A. C. (1963). Deposition of iodine onto plant leaves from air. Health Phys. 9, 1149–1157. 10.1097/00004032-196312000-00011 14086656

[B13] Berg G. Grube M. Schloter M. Smalla K. (2014). Unraveling the plant microbiome: looking back and future perspectives. Front. Microbiol. 5:148. 10.3389/fmicb.2014.00148 24926286 PMC4045152

[B14] Blasco B. Leyva R. Romero L. Ruiz J. M. (2013). Iodine effects on phenolic metabolism in lettuce plants under salt stress. J. Agric. Food Chem. 61, 2591–2596. 10.1021/jf303917n 23445402

[B15] Blasco B. Rios J. J. Cervilla L. M. Sánchez-Rodrigez E. Ruiz J. M. Romero L. (2008). Iodine biofortification and antioxidant capacity of lettuce: potential benefits for cultivation and human health. Ann. Appl. Biol. 152, 289–299. 10.1111/j.1744-7348.2008.00217.x

[B16] Blasco B. Rios J. J. Cervilla L. M. Sánchez-Rodríguez E. Rubio-Wilhelmi M. M. Rosales M. A. (2010). Photorespiration process and nitrogen metabolism in lettuce plants (*Lactuca sativa* L.): induced changes in response to iodine biofortification. J. Plant Growth Regul. 29, 477–486. 10.1007/s00344-010-9159-7

[B17] Blasco B. Ríos J. J. Leyva R. Cervilla L. M. Sánchez-Rodríguez E. Rubio-Wilhelmi M. M.. (2011). Does iodine biofortification affect oxidative metabolism in lettuce plants? Biol. Trace Elem. Res. 142, 831–842. 10.1007/s12011-010-8816-9 20838926

[B18] Borst Pauwels G. W. F. H. (1961). Iodine as a micronutrient for plants. Plant Soil 14, 377–392. 10.1007/BF01666295

[B19] Borst Pauwels G. W. F. H. (1962). An investigation into the effects of iodide and iodate on plant growth. Plant Soil 16, 284–292. 10.1007/BF01381340

[B20] Bowley H. (2013). Iodine Dynamics In The Terrestrial Environment. dissertation/Ph.D. thesis, University of Nottingham, Nottingham. Available online at: http://eprints.nottingham.ac.uk/13241/1/Iodine_dynamics_in_the_terrestrial_environment_-_Hannah_Bowley.pdf (Accessed January 31, 2016).

[B21] Bulloch M. N. (2014). Acute iodine toxicity from a suspected oral methamphetamine ingestion. Clin. Med. Insights Case Rep. 7, 127–129. 10.4137/CCRep.S20086 25452705 PMC4237150

[B22] Burlingame B. (2013). The Role of Agriculture in Diet: Quantity versus Quality no Title. Conf. Front. Agric. Sustain. Stud. Protein Supply Chain to Improv. Diet. Qual. New York Acad. Sci. Available online at: http://www.nyas.org/Publications/Ebriefings/Detail.aspx?cid=5309dc45-a7a8-4611-90f6-dba5b2a139b6 (Accessed January 1, 2016).

[B23] Businelli D. D'Amato R. Onofri A. Tedeschini E. Tei F. (2015). Se-enrichment of cucumber (*Cucumis sativus* L.), lettuce (*Lactuca sativa* L.) and tomato (*Solanum lycopersicum* L. *Karst)* through fortification in pre-transplanting. Sci. Hortic. 197, 697–704. 10.1016/j.scienta.2015.10.039

[B24] Caffagni A. Arru L. Meriggi P. Milc J. Perata P. Pecchioni N. (2011). Iodine fortification plant screening process and accumulation in tomato fruits and potato tubers. Commun. Soil Sci. Plant Anal. 42, 706–718. 10.1080/00103624.2011.550372

[B25] Caffagni A. Pecchioni N. Meriggi P. Bucci V. Sabatini E. Acciarri N. (2012). Iodine uptake and distribution in horticultural and fruit tree species. Ital. J. Agron. 7:32 10.4081/ija.2012.e32

[B26] Cao X.-Y. Jiang X.-M. Dou Z.-H. Rakeman M. A. Kareem A. Zang M.-L.. (1994). Iodination of irrigation water as a method of supplying iodine to a severely iodine-deficient population in XinJiang, China. Lancet 344, 107–110. 10.1016/S0140-6736(94)91286-6 7912349

[B27] Cappuyns V. Swennen R. (2014). Release of vanadium from oxidized sediments: insights from different extraction and leaching procedures. Environ. Sci. Pollut. Res. Int. 21, 2272–2282. 10.1007/s11356-013-2149-0 24057962

[B28] Carpenter L. J. Malin G. Liss P. S. Küpper F. C. (2000). Novel biogenic iodine-containing trihalomethanes and other short-lived halocarbons in the coastal east Atlantic. Global Biogeochem. Cycles 14, 1191–1204. 10.1029/2000GB001257

[B29] Charlton K. Skeaff S. (2011). Iodine fortification: why, when, what, how, and who? Curr. Opin. Clin. Nutr. Metab. Care 14, 618–624. 10.1097/MCO.0b013e32834b2b30 21892078

[B30] Charlton K. E. Jooste P. L. Steyn K. Levitt N. S. Ghosh A. (2013). A lowered salt intake does not compromise iodine status in Cape Town, South Africa, where salt iodization is mandatory. Nutrition 29, 630–634. 10.1016/j.nut.2012.09.010 23274097

[B31] Colmenero-Flores J. M. Martínez G. Gamba G. Vázquez N. Iglesias D. J. Brumós J.. (2007). Identification and functional characterization of cation-chloride cotransporters in plants. Plant J. 50, 278–292. 10.1111/j.1365-313X.2007.03048.x 17355435

[B32] Crockford S. J. (2009). Evolutionary roots of iodine and thyroid hormones in cell-cell signaling. Integr. Comp. Biol. 49, 155–166. 10.1093/icb/icp053 21669854

[B33] Cui X. Sang Y. Song J. (2003). [Residual of exogenous iodine in forest soils and its effect on some wild-vegetable plants]. J. Appl. Ecol. 14, 1612–1616. 14986350

[B34] Dai J. L. Zhang M. Hu Q. H. Huang Y. Z. Wang R. Q. Zhu Y. G. (2009). Adsorption and desorption of iodine by various Chinese soils: II. Iodide and iodate. Geoderma 153, 130–135. 10.1016/j.geoderma.2009.07.020

[B35] Dai J.-L. Zhang M. Zhu Y.-G. (2004a). Adsorption and desorption of iodine by various Chinese soils: I. Iodate. Environ. Int. 30, 525–530. 10.1016/j.envint.2003.10.007 15031012

[B36] Dai J. L. Zhu Y. G. Huang Y. Z. Zhang M. Song J. L. (2006). Availability of iodide and iodate to spinach (*Spinacia oleracea* L.) in relation to total iodine in soil solution. Plant Soil 289, 301–308. 10.1007/s11104-006-9139-7

[B37] Dai J.-L. Zhu Y.-G. Zhang M. Huang Y.-Z. (2004b). Selecting iodine-enriched vegetables and the residual effect of iodate application to soil. Biol. Trace Elem. Res. 101, 265–276. 10.1385/BTER:101:3:265 15564656

[B38] de Benoist B. McLean E. Andersson M. Rogers L. (2008). Iodine deficiency in 2007: global progress since 2003. Food Nutr. Bull. 29, 195–202. 10.1177/156482650802900305 18947032

[B39] de Caffarelli E. (1997). Iodine. Consequences of a deficiency, of excessive iodine, and value of systematic supplementation. J. Gynécol. Obstet. Biol. Reprod. 26, 90–94. 9471472

[B40] Dey(née Pathak) G. Mukherjee R. K. (1984). Iodine treatment of soybean and sunflower seeds for controlling deterioration. Field Crop Res. 9, 205–213. 10.1016/0378-4290(84)90026-1

[B41] Eales J. G. (1997). Iodine metabolism and thyroid-related functions in organisms lacking thyroid follicles: are thyroid hormones also vitamins? Exp. Biol. Med. 214, 302–317. 10.3181/00379727-214-44098 9111521

[B42] Eneqvist T. Lundberg E. Nilsson L. Abagyan R. Sauer-Eriksson A. E. (2003). The transthyretin-related protein family. Eur. J. Biochem. 270, 518–532. 10.1046/j.1432-1033.2003.03408.x 12542701

[B43] FAO (2009). The State of Food Insecurity in the World. Rome: Food and Agriculture Organization of the United Nations.

[B44] Fordyce F. M. (2003). Database of the Iodine Content of Food and Diets Populated with Data from Published Literature. Commisioned Report CR/03/84N. Nottingham: British Geological Survey.

[B45] Fournier J.-B. Rebuffet E. Delage L. Grijol R. Meslet-Cladière L. Rzonca J.. (2014). The Vanadium Iodoperoxidase from the marine flavobacteriaceae species Zobellia galactanivorans reveals novel molecular and evolutionary features of halide specificity in the vanadium haloperoxidase enzyme family. Appl. Environ. Microbiol. 80, 7561–7573. 10.1128/AEM.02430-14 25261522 PMC4249250

[B46] Fricke W. (2015). The significance of water co-transport for sustaining transpirational water flow in plants: a quantitative approach. J. Exp. Bot. 66, 731–739. 10.1093/jxb/eru466 25563967

[B47] Fuge R. (1996). Geochemistry of iodine in relation to iodine deficiency diseases. Geol. Soc. 113, 201–211. 10.1144/GSL.SP.1996.113.01.16

[B48] Fuge R. (2005). Soils and iodine deficiency, in Essentials of Medical Geology, eds Selinus O. Alloway B. Centeno J. A. Finkelman R. B. Fuge R. Lindh U. Smedley P. (New York, NY: Elsevier Academic Press), 417–433.

[B49] Fuge R. (2013). Soils and iodine deficiency, in Essentials of Medical Geology: Revised Edition, ed Selinus O. (Dordrecht: Springer Netherlands), 417–432.

[B50] Fuge R. Johnson C. C. (1986). The geochemistry of iodine - a review. Environ. Geochem. Health 8, 31–54. 10.1007/BF02311063 24213950

[B51] Fuge R. Johnson C. C. (2015). Iodine and human health, the role of environmental geochemistry and diet, a review. Appl. Geochem. 63, 282–302. 10.1016/j.apgeochem.2015.09.013

[B52] Funahashi H. Imai T. Mase T. Sekiya M. Yokoi K. Hayashi H.. (2001). Seaweed prevents breast cancer? Jpn. J. Cancer Res. 92, 483–487. 10.1111/j.1349-7006.2001.tb01119.x 11376555 PMC5926746

[B53] García-Osuna H. T. Benavides-Mendoza A. Rivas-Morales C. Morales-Rubio E. Verde-Star J. Miranda-Ruvalcaba R. (2014). Iodine application increased ascorbic acid content and modified the vascular tissue in Opuntia ficus-indica. Pak. J. Bot. 46, 127–134. Available online at: http://www.pakbs.org/pjbot/abstracts/46(1)/13.html

[B54] García-Solís P. Alfaro Y. Anguiano B. Delgado G. Guzman R. C. Nandi S.. (2005). Inhibition of N-methyl-N-nitrosourea-induced mammary carcinogenesis by molecular iodine (I2) but not by iodide (I-) treatment Evidence that I2 prevents cancer promotion. Mol. Cell. Endocrinol. 236, 49–57. 10.1016/j.mce.2005.03.001 15922087

[B55] Gattullo C. E. Allegretta I. Medici L. Fijan R. Pii Y. Cesco S. (2016). Silicon dynamics in the rhizosphere: connections with iron mobilization. J. Plant Nutr. Soil Sci. 179, 409–417. 10.1002/jpln.201500535

[B56] Gill S. S. Tuteja N. (2010). Reactive oxygen species and antioxidant machinery in abiotic stress tolerance in crop plants. Plant Physiol. Biochem. 48, 909–930. 10.1016/j.plaphy.2010.08.016 20870416

[B57] Gniwotta F. Vogg G. Gartmann V. Carver T. L. W. Riederer M. Jetter R. (2005). What do microbes encounter at the plant surface? Chemical composition of pea leaf cuticular waxes. Plant Physiol. 139, 519–530. 10.1104/pp.104.053579 16113231 PMC1203400

[B58] Guido-Garcia F. Law G. T. W. Lloyd J. R. Lythgoe P. Morris K. (2015). Bioreduction of iodate in sediment microcosms. Mineral. Mag. 79, 1343–1351. 10.1180/minmag.2015.079.6.10

[B59] Gupta N. Bajpai M. Majumdar R. Mishra P. (2015). Response of iodine on antioxidant levels of Glycine max L. Grown under Cd2+ stress. Adv. Biol. Res. (Rennes). 9, 40–48. 10.5829/idosi.abr.2015.9.1.9183

[B60] Gupta U. C. Gupta S. C. (1998). Trace element toxicity relationships to crop production and livestock and human health: implications for management. Commun. Soil Sci. Plant Anal. 29, 1491–1522. 10.1080/00103629809370045

[B61] Hageman R. H. Hodge E. S. McHargue J. S. (1942). Effect of potassium iodide on the ascorbic acid content and growth of tomato plants. Plant Physiol. 17, 465–472. 10.1104/pp.17.3.465 16653793 PMC438041

[B62] He X. Nara K. (2007). Element biofortification: can mycorrhizas potentially offer a more effective and sustainable pathway to curb human malnutrition? Trends Plant Sci. 12, 331–333. 10.1016/j.tplants.2007.06.008 17658289

[B63] Henner P. Hurtevent P. Thiry Y. Levchuk S. Yoschenko V. Kashparov V. (2013). Translocation of (125)I, (75)Se and (36)Cl to edible parts of radish, potato and green bean following wet foliar contamination under field conditions. J. Environ. Radioact. 124, 171–184. 10.1016/j.jenvrad.2013.05.012 23811127

[B64] Herrett R. A. Hatfield H. H. Crosby D. G. Vlitos A. J. (1962). Leaf abscission induced by the iodide ion. Plant Physiol. 37, 358–363. 10.1104/pp.37.3.358 16655658 PMC549793

[B65] Heyland A. Moroz L. L. (2005). Cross-kingdom hormonal signaling: an insight from thyroid hormone functions in marine larvae. J. Exp. Biol. 208, 4355–4361. 10.1242/jeb.01877 16339856

[B66] Hong C. Weng H. Jilani G. Yan A. Liu H. Xue Z. (2012). Evaluation of iodide and iodate for adsorption-desorption characteristics and bioavailability in three types of soil. Biol. Trace Elem. Res. 146, 262–271. 10.1007/s12011-011-9231-6 22038267

[B67] Hong C.-L. Weng H.-X. Qin Y.-C. Yan A.-L. Xie L.-L. (2008). Transfer of iodine from soil to vegetables by applying exogenous iodine. Agron. Sustain. Dev. 28, 575–583. 10.1051/agro:2008033

[B68] Hurtevent P. Thiry Y. Levchuk S. Yoschenko V. Henner P. Madoz-Escande C.. (2013). Translocation of 125I, 75Se and 36Cl to wheat edible parts following wet foliar contamination under field conditions. J. Environ. Radioact. 121, 43–54. 10.1016/j.jenvrad.2012.04.013 22608977

[B69] Itoh N. Toda H. Matsuda M. Negishi T. Taniguchi T. Ohsawa N. (2009). Involvement of S-adenosylmethionine-dependent halide/thiol methyltransferase (HTMT) in methyl halide emissions from agricultural plants: isolation and characterization of an HTMT-coding gene from Raphanus sativus (daikon radish). BMC Plant Biol. 9:116. 10.1186/1471-2229-9-116 19723322 PMC2752461

[B70] Jajda H. M. Thakkar V. R. (2012). Control of *Aspergillus niger* infection in varieties of *Arachis hypogeae* L. by supplementation of zinc ions during seed germination. Arch. Phytopathol. Plant Prot. 45, 1464–1478. 10.1080/03235408.2012.677312

[B71] Johnson C. C. (2003). The Geochemistry of Iodine and its Application to Environmental Strategies for Reducing the Risks from Iodine Deficiency Disorders (IDD). Nottingham: British Geological Survey.

[B72] Jones C. E. Hornsby K. E. Sommariva R. Dunk R. M. von Glasow R. McFiggans G. (2010). Quantifying the contribution of marine organic gases to atmospheric iodine. Geophys. Res. Lett. 37, 1–6. 10.1029/2010gl043990

[B73] Jones D. L. (1998). Organic acids in the rhizosphere – a critical review. Plant Soil 205, 25–44. 10.1023/A:1004356007312

[B74] Kaplan D. I. Denham M. E. Zhang S. Yeager C. Xu C. Schwehr K. A.. (2014). Radioiodine biogeochemistry and prevalence in groundwater. Crit. Rev. Environ. Sci. Technol. 44, 2287–2335. 10.1080/10643389.2013.828273 25264421 PMC4160254

[B75] Karnani M. Annila A. (2009). Gaia again. Biosystems 95, 82–87. 10.1016/j.biosystems.2008.07.003 18706969

[B76] Kato S. Wachi T. Yoshihira K. Nakagawa T. Ishikawa A. Takagi D.. (2013). Rice (*Oryza sativa* L.) roots have iodate reduction activity in response to iodine. Front. Plant Sci. 4:227. 10.3389/fpls.2013.00227 23847633 PMC3706741

[B77] Kiferle C. Gonzali S. Holwerda H. T. Real Ibaceta R. Perata P. (2013). Tomato fruits: a good target for iodine biofortification. Front. Plant Sci. 4:205. 10.3389/fpls.2013.00205 23818889 PMC3694224

[B78] Klebanoff S. J. (1982). The iron-H2O2-iodide cytotoxic system. J. Exp. Med. 156, 1262–1267. 10.1084/jem.156.4.1262 6296262 PMC2186828

[B79] Kopeć A. Piątkowska E. Bieżanowska-Kopeć R. Pysz M. Koronowicz A. Kapusta-Duch J. (2015). Effect of lettuce biofortified with iodine by soil fertilization on iodine concentration in various tissues and selected biochemical parameters in serum of Wistar rats. J. Funct. Foods 14, 479–486. 10.1016/j.jff.2015.02.027

[B80] Kotwal A. Priya R. Qadeer I. (2007). Goiter and other iodine deficiency disorders: a systematic review of epidemiological studies to deconstruct the complex web. Arch. Med. Res. 38, 1–14. 10.1016/j.arcmed.2006.08.006 17174717

[B81] Küpper F. C. Carpenter L. J. McFiggans G. B. Palmer C. J. Waite T. J. Boneberg E.-M.. (2008). Iodide accumulation provides kelp with an inorganic antioxidant impacting atmospheric chemistry. Proc. Natl. Acad. Sci. U.S.A. 105, 6954–6958. 10.1073/pnas.0709959105 18458346 PMC2383960

[B82] Küpper F. C. Feiters M. C. Olofsson B. Kaiho T. Yanagida S. Zimmermann M. B.. (2011). Commemorating two centuries of iodine research: an interdisciplinary overview of current research. Angew. Chem. Int. Ed. Engl. 50, 11598–11620. 10.1002/anie.201100028 22113847

[B83] Küpper F. C. Schweigert N. Ar Gall E. Legendre J.-M. Vilter H. Kloareg B. (1998). Iodine uptake in Laminariales involves extracellular, haloperoxidase-mediated oxidation of iodide. Planta 207, 163–171. 10.1007/s004250050469

[B84] Landini M. Gonzali S. Kiferle C. Tonacchera M. Agretti P. Dimida A.. (2012). Metabolic engineering of the iodine content in Arabidopsis. Sci. Rep. 2:338. 10.1038/srep00338 22468225 PMC3313481

[B85] Landini M. Gonzali S. Perata P. (2011). Iodine biofortification in tomato. J. Plant Nutr. Soil Sci. 174, 480–486. 10.1002/jpln.201000395

[B86] Lawson P. G. (2014). Development and Evaluation of Iodine Biofortification Strategies for Vegetables. Berlin: Logos Verlag Berlin GmbH.

[B87] Lawson P. G. Daum D. Czauderna R. Meuser H. Härtling J. W. (2015). Soil versus foliar iodine fertilization as a biofortification strategy for field-grown vegetables. Front. Plant Sci. 6:450 10.3389/fpls.2015.00450 PMC4477264 26157445

[B88] Lazarus J. H. Bestwick J. P. Channon S. Paradice R. Maina A. Rees R.. (2012). Antenatal thyroid screening and childhood cognitive function. N. Engl. J. Med. 366, 493–501. 10.1056/NEJMoa1106104 22316443 PMC3814060

[B89] Leblanc C. Colin C. Cosse A. Delage L. La Barre S. Morin P.. (2006). Iodine transfers in the coastal marine environment: the key role of brown algae and of their vanadium-dependent haloperoxidases. Biochimie 88, 1773–1785. 10.1016/j.biochi.2006.09.001 17007992

[B90] Lehr J. J. Wybenga J. M. Rosanow M. (1958). Iodine as a micronutrient for tomatoes. Plant Physiol. 33, 421–427. 10.1104/pp.33.6.421 16655161 PMC541118

[B91] Leri A. C. Ravel B. (2015). Abiotic bromination of soil organic matter. Environ. Sci. Technol. 49, 13350–13359. 10.1021/acs.est.5b03937 26468620 PMC4950848

[B92] Leung H.-M. Wang Z.-W. Ye Z.-H. Yung K.-L. Peng X.-L. Cheung K.-C. (2013). Interactions between arbuscular mycorrhizae and plants in phytoremediation of metal-contaminated soils: a review. Pedosphere 23, 549–563. 10.1016/S1002-0160(13)60049-1

[B93] Leyva R. Sánchez-Rodríguez E. Ríos J. J. Rubio-Wilhelmi M. M. Romero L. Ruiz J. M.. (2011). Beneficial effects of exogenous iodine in lettuce plants subjected to salinity stress. Plant Sci. 181, 195–202. 10.1016/j.plantsci.2011.05.007 21683885

[B94] Li H.-P. Daniel B. Creeley D. Grandbois R. Zhang S. Xu C.. (2014). Superoxide production by a manganese-oxidizing bacterium facilitates iodide oxidation. Appl. Environ. Microbiol. 80, 2693–2699. 10.1128/AEM.00400-14 24561582 PMC3993295

[B95] Li H.-P. Yeager C. M. Brinkmeyer R. Zhang S. Ho Y.-F. Xu C.. (2012). Bacterial production of organic acids enhances H2O2-dependent iodide oxidation. Environ. Sci. Technol. 46, 4837–4844. 10.1021/es203683v 22455542

[B96] Li R. Liu H.-P. Hong C.-L. Dai Z.-X. Liu J.-W. Zhou J.. (2016). Iodide and iodate effects on the growth and fruit quality of strawberry. J. Sci. Food Agric. 10.1002/jsfa.7719. [Epub ahead of print]. 26992053

[B97] Limchoowong N. Sricharoen P. Techawongstien S. Chanthai S. (2016). An iodine supplementation of tomato fruits coated with an edible film of the iodide-doped chitosan. Food Chem. 200, 223–229. 10.1016/j.foodchem.2016.01.042 26830582

[B98] Longvah T. Toteja G. S. Bulliyya G. Raghuvanshi R. S. Jain S. Rao V. (2012). Stability of added iodine in different Indian cooking processes. Food Chem. 130, 953–959. 10.1016/j.foodchem.2011.08.024

[B99] Ma Q. Bao A.-K. Chai W.-W. Wang W.-Y. Zhang J.-L. Li Y.-X. (2016). Transcriptomic analysis of the succulent xerophyte Zygophyllum xanthoxylum in response to salt treatment and osmotic stress. Plant Soil 402, 343–361. 10.1007/s11104-016-2809-1

[B100] Mackowiak C. L. Grossl P. R. (1999). Iodate and iodide effects on iodine uptake and partitioning in rice (*Oryza sativa* L.) grown in solution culture. Plant Soil 212, 133–141. 10.1023/A:1004666607330 11762382

[B101] Mackowiak C. L. Grossl P. R. Cook K. L. (2005). Iodine toxicity in a plant-solution system with and without humic acid. Plant Soil 269, 141–150. 10.1007/s11104-004-0401-6

[B102] Mao H. Wang J. Wang Z. Zan Y. Lyons G. Zou C. (2014). Using agronomic biofortification to boost zinc, selenium, and iodine concentrations of food crops grown on the loess plateau in China. J. Soil Sci. Plant Nutr. 14, 459–470. 10.4067/s0718-95162014005000036

[B103] McFiggans G. Coe H. Burgess R. Allan J. Cubison M. Alfarra M. R. (2004). Direct evidence for coastal iodine particles from Laminaria macroalgae – linkage to emissions of molecular iodine. Atmos. Chem. Phys. 4, 701–713. 10.5194/acp-4-701-2004

[B104] Miller A. E. M. Heyland A. (2013). Iodine accumulation in sea urchin larvae is dependent on peroxide. J. Exp. Biol. 216, 915–926. 10.1242/jeb.077958 23155081

[B105] Miyake Y. Tsunogai S. (1963). Evaporation of iodine from the ocean. J. Geophys. Res. 68, 3989–3993. 10.1029/JZ068i013p03989

[B106] Moore R. M. Groszko W. (1999). Methyl iodide distribution in the ocean and fluxes to the atmosphere. J. Geophys. Res. Ocean. 104, 11163–11171. 10.1029/1998JC900073

[B107] Moreda-Piñeiro A. Romarís-Hortas V. Bermejo-Barrera P. (2011). A review on iodine speciation for environmental, biological and nutrition fields. J. Anal. At. Spectrom. 26, 2107–2152. 10.1039/C0JA00272K

[B108] Mottiar Y. (2013). Iodine biofortification through plant biotechnology. Nutrition 29, 1431. 10.1016/j.nut.2013.04.009 23948340

[B109] Mottiar Y. Altosaar I. (2011). Iodine sequestration by amylose to combat iodine deficiency disorders. Trends Food Sci. Technol. 22, 335–340. 10.1016/j.tifs.2011.02.007

[B110] Moyers J. L. Duce R. A. (1972). Gaseous and particulate iodine in the marine atmosphere. J. Geophys. Res. 77, 5229–5238. 10.1029/JC077i027p05229

[B111] Muramatsu Y. Christoffers D. Ohmomo Y. (1983). Influence of chemical forms on iodine uptake by plant. J. Radiat. Res. 24, 326–338. 10.1269/jrr.24.326 6676469

[B112] Muramatsu Y. Yoshida S. (1995). Volatilization of methyl iodide from the soil-plant system. Atmos. Environ. 29, 21–25. 10.1016/1352-2310(94)00220-F

[B113] Muramatsu Y. Yoshida S. (1999). Effects of microorganisms on the fate of iodine in the soil environment. Geomicrobiol. J. 16, 85–93. 10.1080/014904599270776

[B114] Nakamura Y. Ohmomo Y. (1984). Transfer of gaseous iodine to Tradescantia. J. Radiat. Res. 25, 251–259. 10.1269/jrr.25.251 6512747

[B115] Nakamura Y. Sumiya M. Uchida S. Ohmomo Y. (1986). Transfer of gaseous iodine to rice plants. J. Radiat. Res. 27, 171–182. 10.1269/jrr.27.171 3795163

[B116] Nguyen C. T. Agorio A. Jossier M. Depré S. Thomine S. Filleur S. (2016). Characterization of the chloride channel-like, atclcg, involved in chloride tolerance in *Arabidopsis thaliana*. Plant Cell Physiol. 57, 764–775. 10.1093/pcp/pcv169 26556649

[B117] Nitschke U. Dixneuf S. Ruth A. A. Schmid M. Stengel D. B. (2013). Molecular iodine (I2) emission from two Laminaria species (Phaeophyceae) and impact of irradiance and temperature on I2 emission into air and iodide release into seawater from *Laminaria digitata*. Mar. Environ. Res. 92, 102–109. 10.1016/j.marenvres.2013.09.006 24080409

[B118] Pearce E. N. Andersson M. Zimmermann M. B. (2013). Global iodine nutrition: where do we stand in 2013? Thyroid 23, 523–528. 10.1089/thy.2013.0128 23472655

[B119] Pellegrino E. Bedini S. (2014). Enhancing ecosystem services in sustainable agriculture: biofertilization and biofortification of chickpea (*Cicer arietinum* L.) by arbuscular mycorrhizal fungi. Soil Biol. Biochem. 68, 429–439. 10.1016/j.soilbio.2013.09.030

[B120] Pennington J. A. (1990). A review of iodine toxicity reports. J. Am. Diet. Assoc. 90, 1571–1581. 2229854

[B121] Pessoa J. Sárkány Z. Ferreira-da-Silva F. Martins S. Almeida M. R. Li J.. (2010). Functional characterization of Arabidopsis thaliana transthyretin-like protein. BMC Plant Biol. 10:30. 10.1186/1471-2229-10-30 20167108 PMC2834698

[B122] Pii Y. Cesco S. Mimmo T. (2015). Shoot ionome to predict the synergism and antagonism between nutrients as affected by substrate and physiological status. Plant Physiol. Biochem. 94, 48–56. 10.1016/j.plaphy.2015.05.002 26004913

[B123] Pilbeam D. J. Drihem K. (2007). Vanadium, in Handbook of Plant Nutrition, eds Barker A. V. Pilbeam D. J. (Boca Raton, FL: CRC Press), 585–596.

[B124] Porras-Alfaro A. Bayman P. (2011). Hidden fungi, emergent properties: endophytes and microbiomes. Annu. Rev. Phytopathol. 49, 291–315. 10.1146/annurev-phyto-080508-081831 19400639

[B125] Powell A. A. Corbineau F. Franca-Neto J. Léchappé J. Mesterhazy A. Noli E. (2005). Towards the future in seed production, evaluation and improvement. Seed Sci. Technol. 33, 265–281. 10.15258/sst.2005.33.2.01

[B126] Prasad A. S. (2013). Discovery of human zinc deficiency: its impact on human health and disease. Adv. Nutr. 4, 176–190. 10.3945/an.112.003210 23493534 PMC3649098

[B127] Rana A. Kabi S. R. Verma S. Adak A. Pal M. Shivay Y. S. (2015). Prospecting plant growth promoting bacteria and cyanobacteria as options for enrichment of macro- and micronutrients in grains in rice–wheat cropping sequence. Cogent Food Agric. 1, 1–16. 10.1080/23311932.2015.1037379

[B128] Redeker K. R. Andrews J. Fisher F. Sass R. Cicerone R. J. (2002). Interfield and intrafield variability of methyl halide emissions from rice paddies. Global Biogeochem. Cycles 16, 72-1–72-9. 10.1029/2002GB001874

[B129] Redeker K. R. Manley S. L. Walser M. Cicerone R. J. (2004). Physiological and biochemical controls over methyl halide emissions from rice plants. Global Biogeochem. Cycles 18, 1–14. 10.1029/2003gb002042

[B130] Reiller P. Mercier-Bion F. Gimenez N. Barré N. Miserque F. (2006). Iodination of humic acid samples from different origins. Radiochim. Acta 94, 739–745. 10.1524/ract.2006.94.9-11.739

[B131] Ren Q. Fan J. Zhang Z. Zheng X. Delong G. R. (2008). An environmental approach to correcting iodine deficiency: supplementing iodine in soil by iodination of irrigation water in remote areas. J. Trace Elem. Med. Biol. 22, 1–8. 10.1016/j.jtemb.2007.09.003 18319134

[B132] Roberts S. K. (2006). Plasma membrane anion channels in higher plants and their putative functions in roots. New Phytol. 169, 647–666. 10.1111/j.1469-8137.2006.01639.x 16441747

[B133] Rose N. R. Bonita R. Burek C. L. (2002). Iodine: an environmental trigger of thyroiditis. Autoimmun. Rev. 1, 97–103. 10.1016/S1568-9972(01)00016-7 12849065

[B134] Runia W. T. H. (1995). A review of possibilities for disinfection of recirculation water from soilless cultures. Acta Hortic. 382, 221–229. 10.17660/ActaHortic.1995.382.25

[B135] Saini H. S. Attieh J. M. Hanson A. D. (1995). Biosynthesis of halomethanes and methanethiol by higher plants via a novel methyltransferase reaction. Plant Cell Environ. 18, 1027–1033. 10.1111/j.1365-3040.1995.tb00613.x

[B136] Saunders R. W. Kumar R. MacDonald S. M. Plane J. M. C. (2012). Insights into the photochemical transformation of iodine in aqueous systems: humic acid photosensitized reduction of iodate. Environ. Sci. Technol. 46, 11854–11861. 10.1021/es3030935 23038990

[B137] Saunders R. W. Plane J. M. C. (2005). Formation pathways and composition of iodine oxide ultra-fine particles. Environ. Chem. 2, 299–303. 10.1071/EN05079

[B138] Schlorke D. Flemmig J. Birkemeyer C. Arnhold J. (2016). Formation of cyanogen iodide by lactoperoxidase. J. Inorg. Biochem. 154, 35–41. 10.1016/j.jinorgbio.2015.11.005 26580225

[B139] Seki M. Oikawa J. Taguchi T. Ohnuki T. Muramatsu Y. Sakamoto K.. (2013). Laccase-catalyzed oxidation of iodide and formation of organically bound iodine in soils. Environ. Sci. Technol. 47, 390–397. 10.1021/es303228n 23194146

[B140] Shabala S. (2013). Learning from halophytes: physiological basis and strategies to improve abiotic stress tolerance in crops. Ann. Bot. 112, 1209–1221. 10.1093/aob/mct205 24085482 PMC3806534

[B141] Shaw G. Scott L. K. Kinnersley R. P. (2007). Sorption of caesium, iodine and sulphur in solution to the adaxial leaf surface of broad bean (*Vicia faba* L.). Environ. Exp. Bot. 59, 361–370. 10.1016/j.envexpbot.2006.04.008

[B142] Sheppard M. I. Thibault D. H. (1992). Chemical behaviour of iodine in organic and mineral soils. Appl. Geochem. 7, 265–272. 10.1016/0883-2927(92)90042-2

[B143] Sheppard M. I. Thibault D. H. Smith P. A. Hawkins J. L. (1994). Volatilization: a soil degassing coefficient for iodine. J. Environ. Radioact. 25, 189–203. 10.1016/0265-931X(94)90072-8

[B144] Shetaya W. H. Young S. D. Watts M. J. Ander E. L. Bailey E. H. (2012). Iodine dynamics in soils. Geochim. Cosmochim. Acta 77, 457–473. 10.1016/j.gca.2011.10.034

[B145] Shimamoto Y. S. Takahashi Y. Terada Y. (2011). Formation of organic iodine supplied as iodide in a soil-water system in Chiba, Japan. Environ. Sci. Technol. 45, 2086–2092. 10.1021/es1032162 21322630

[B146] Shinonaga T. Gerzabek M. Strebl F. Muramatsu Y. (2001). Transfer of iodine from soil to cereal grains in agricultural areas of Austria. Sci. Total Environ. 267, 33–40. 10.1016/S0048-9697(00)00764-6 11286214

[B147] Shiroyama K. Kawasaki Y. Unno Y. Amachi S. (2015). A putative multicopper oxidase, IoxA, is involved in iodide oxidation by Roseovarius sp. strain A-2. Biosci. Biotechnol. Biochem. 79, 1898–905. 10.1080/09168451.2015.1052767 26041311

[B148] Silva-Moreno E. Brito-Echeverría J. López M. Ríos J. Balic I. Campos-Vargas R.. (2016). Effect of cuticular waxes compounds from table grapes on growth, germination and gene expression in Botrytis cinerea. World J. Microbiol. Biotechnol. 32, 74. 10.1007/s11274-016-2041-4 27038944

[B149] Singh A. K. Singh A. Singh A. K. Shamim M. Vikram P. Singh S. (2012). Application of potassium iodide as a new agent for screening of drought tolerance upland rice genotypes at flowering stage. Plant Knowl. J. 1, 25–32. Available online at: https://search.informit.com.au/documentSummary;dn=939538551491567;res=IELENG

[B150] Smoleń S. Kowalska I. Sady W. (2014a). Assessment of biofortification with iodine and selenium of lettuce cultivated in the NFT hydroponic system. Sci. Hortic. 166, 9–16. 10.1016/j.scienta.2013.11.011

[B151] Smoleń S. Ledwożyw-Smoleń I. Sady W. (2015a). The role of exogenous humic and fulvic acids in iodine biofortification in spinach (*Spinacia oleracea* L.). Plant Soil 1–15. 10.1007/s11104-015-2785-x

[B152] Smoleń S. Rozek R. Ledwozyw-Smolen I. Strzetelski P. (2011). Preliminary evaluation of the nfluence of soil fertilization and foliar nutrition with iodine on the efficiency of iodine biofortification and chemical composition of lettuce. J. Elem. 16, 613–622. 10.5601/jelem.2011.16.4.10

[B153] Smoleń S. Sady W. (2012). Influence of iodine form and application method on the effectiveness of iodine biofortification, nitrogen metabolism as well as the content of mineral nutrients and heavy metals in spinach plants (*Spinacia oleracea* L.). Sci. Hortic. 143, 176–183. 10.1016/j.scienta.2012.06.006

[B154] Smoleń S. Sady W. Ledwożyw-Smoleń I. Strzetelski P. Liszka-Skoczylas M. Rożek S. (2014b). Quality of fresh and stored carrots depending on iodine and nitrogen fertilization. Food Chem. 159, 316–322. 10.1016/j.foodchem.2014.03.024 24767061

[B155] Smoleń S. Skoczylas Ł. Rakoczy R. Ledwożyw-Smolen I. Kopec A. Piatkowska E. (2015b). Mineral composition of field-grown lettuce (*Lactuca sativa* L.) depending on the diversified fertilization with iodine and selenium compounds. Acta Sci. Pol. Hortorum Cultus 14, 97–114. Available online at: http://www.acta.media.pl/pl/full/7/2015/000070201500014000060009700114.pdf

[B156] Smoleń S. Wierzbińska J. Sady W. Kołton A. Wiszniewska A. Liszka-Skoczylas M. (2015c). Iodine biofortification with additional application of salicylic acid affects yield and selected parameters of chemical composition of tomato fruits (*Solanum lycopersicum* L.). Sci. Hortic. 188, 89–96. 10.1016/j.scienta.2015.03.023

[B157] Stavber S. Jereb M. Zupan M. (2008). Electrophilic iodination of organic compounds using elemental iodine or iodides. Synthesis 2008, 1487–1513. 10.1055/s-2008-106703727505233

[B158] Steinberg S. Kimble G. Schmett G. Emerson D. Turner M. Rudin M. (2008). Abiotic reaction of iodate with sphagnum peat and other natural organic matter. J. Radioanal. Nucl. Chem. 277, 185–191. 10.1007/s10967-008-0728-1

[B159] Steinnes E. (2009). Soils and geomedicine. Environ. Geochem. Health 31, 523–535. 10.1007/s10653-009-9257-2 19350398

[B160] Stewart A. G. Carter J. Parker A. Alloway B. J. (2003). The illusion of environmental iodine deficiency. Environ. Geochem. Health 25, 165–170. 10.1023/A:1021281822514 12901092

[B161] Strzetelski P. Smoleń S. Rozek S. Sady W. (2010). The effect of diverse iodine fertilization on nitrate accumulation and content of selected compounds in radish plants (*Raphanus sativus* L.). Acta Sci. Pol. Hortorum Cultus 9, 65–73. Available online at: https://www.infona.pl/resource/bwmeta1.element.dl-catalog-15502fac-cba9-4081-b367-847faf1bc05f

[B162] Sun W. Q. Leopold A. C. (1995). The Maillard reaction and oxidative stress during aging of soybean seeds. Physiol. Plant. 94, 94–104. 10.1111/j.1399-3054.1995.tb00789.x

[B163] Suzuki M. Eda Y. Ohsawa S. Kanesaki Y. Yoshikawa H. Tanaka K.. (2012). Iodide oxidation by a novel multicopper oxidase from the alphaproteobacterium strain Q-1. Appl. Environ. Microbiol. 78, 3941–3949. 10.1128/AEM.00084-12 22447601 PMC3346402

[B164] Takekawa Y. Nakamura T. (2012). Rice OsHOL1 and OsHOL2 proteins have S-adenosyl-L-methionine-dependent methyltransferase activities toward iodide ions. Plant Biotechnol. 29, 103–108. 10.5511/plantbiotechnology.12.0207a

[B165] Terzano R. Cesco S. Mimmo T. (2015). Dynamics, thermodynamics and kinetics of exudates: crucial issues in understanding rhizosphere processes. Plant Soil 386, 399–406. 10.1007/s11104-014-2308-1

[B166] Tschiersch J. Shinonaga T. Heuberger H. (2009). Dry deposition of gaseous radioiodine and particulate radiocaesium onto leafy vegetables. Sci. Total Environ. 407, 5685–5693. 10.1016/j.scitotenv.2009.06.025 19640563

[B167] Tsukada H. Takeda A. Tagami K. Uchida S. (2008). Uptake and distribution of iodine in rice plants. J. Environ. Qual. 37, 2243–2247. 10.2134/jeq2008.0010 18948477

[B168] Ujowundu C. Ukoha A. Agha C. Nwachukwu N. Igwe K. Kalu F. (2010). Effects of potassium iodate application on the biomass and iodine concentration of selected indigenous nigerian vegetables. Afr. J. Biotechnol. 9, 7141–7147. 10.4314/ajb.v9i42

[B169] Umaly R. C. Poel L. W. (1971). Effects of lodine in various formulations on the growth of barley and pea plants in nutrient solution culture. Ann. Bot. 35, 127–131.

[B170] Venturi S. (2011). Evolutionary significance of iodine. Curr. Chem. Biol. 5, 155–162. 10.2174/2212796811105030155

[B171] Vinogradov A. P. Lapp M. A. (1971). Use of iodine haloes to search for concealed mineralisation. Vestn. -Lemngradskii Univ. Seriia Geol. Geogr. 24, 70–76.

[B172] Vought R. L. Brown F. A. London W. T. (1970). Iodine in the environment. Arch. Environ. Heal. An Int. J. 20, 516–522. 10.1080/00039896.1970.10665632 5429991

[B173] Voogt W. Holwerda H. T. Khodabaks R. (2010). Biofortification of lettuce (*Lactuca sativa* L.) with iodine: the effect of iodine form and concentration in the nutrient solution on growth, development and iodine uptake of lettuce grown in water culture. J. Sci. Food Agric. 90, 906–913. 10.1002/jsfa.3902 20355129

[B174] Voogt W. Jackson W. A. (2010). Perchlorate, nitrate, and iodine uptake and distribution in lettuce (*Lactuca sativa* L.) and potential impact on background levels in humans. J. Agric. Food Chem. 58, 12192–12198. 10.1021/jf101227d 21047133

[B175] Watts M. J. O'Reilly J. Maricelli A. Coleman A. Ander E. L. Ward N. I. (2010). A snapshot of environmental iodine and selenium in La Pampa and San Juan provinces of Argentina. J. Geochem. Explor. 107, 87–93. 10.1016/j.gexplo.2009.11.002

[B176] Wegner L. H. (2014). Root pressure and beyond: energetically uphill water transport into xylem vessels? J. Exp. Bot. 65, 381–393. 10.1093/jxb/ert391 24311819

[B177] Weng H. Hong C. Xia T. Bao L. Liu H. Li D. (2013). Iodine biofortification of vegetable plants—An innovative method for iodine supplementation. Chin. Sci. Bull. 58, 2066–2072. 10.1007/s11434-013-5709-2

[B178] Weng H.-X. Hong C.-L. Yan A.-L. Pan L.-H. Qin Y.-C. Bao L.-T.. (2008a). Mechanism of iodine uptake by cabbage: effects of iodine species and where it is stored. Biol. Trace Elem. Res. 125, 59–71. 10.1007/s12011-008-8155-2 18521548

[B179] Weng H.-X. Liu H.-P. Li D.-W. Ye M. Pan L. Xia T.-H. (2014). An innovative approach for iodine supplementation using iodine-rich phytogenic food. Environ. Geochem. Health 36, 815–828. 10.1007/s10653-014-9597-4 24504625

[B180] Weng H.-X. Weng J.-K. Yan A.-L. Hong C.-L. Yong W.-B. Qin Y.-C. (2008b). Increment of iodine content in vegetable plants by applying iodized fertilizer and the residual characteristics of iodine in soil. Biol. Trace Elem. Res. 123, 218–228. 10.1007/s12011-008-8094-y 18265951

[B181] Weng H.-X. Weng J.-K. Yong W.-B. Sun X.-W. Zhong H. (2003). Capacity and degree of iodine absorbed and enriched by vegetable from soil. J. Environ. Sci. (China) 15, 107–111. 12602613

[B182] Weng H.-X. Yan A.-L. Hong C.-L. Xie L.-L. Qin Y.-C. Cheng C. Q. (2008c). Uptake of different species of iodine by water spinach and its effect to growth. Biol. Trace Elem. Res. 124, 184–194. 10.1007/s12011-008-8137-4 18449478

[B183] Wever R. (2012). Structure and function of vanadium haloperoxidases, in Vanadium: Biochemical and Molecular Biological Approaches, ed Michibata H. (Dordrecht: Springer), 97–125.

[B184] White P. J. Broadley M. R. (2009). Biofortification of crops with seven mineral elements often lacking in human diets–iron, zinc, copper, calcium, magnesium, selenium and iodine. New Phytol. 182, 49–84. 10.1111/j.1469-8137.2008.02738.x 19192191

[B185] Whitehead D. C. (1973). Uptake and distribution of iodine in grass and clover plants grown in solution culture. J. Sci. Food Agric. 24, 43–50. 10.1002/jsfa.2740240108 4696594

[B186] Whitehead D. C. (1974). The influence of organic matter, chalk, and sesquioxides on the solubility of iodide, elemental iodine, and iodate incubated with soil. J. Soil Sci. 25, 461–470. 10.1111/j.1365-2389.1974.tb01141.x

[B187] Whitehead D. C. (1978). Iodine in soil profiles in relation to iron and aluminium oxides and organic matter. J. Soil Sci. 29, 88–94. 10.1111/j.1365-2389.1978.tb02035.x

[B188] Whitehead D. C. (1979). Iodine in the U.K. environment with particular reference to agriculture. J. Appl. Ecol. 16, 269–279. 10.2307/2402746

[B189] Whitehead D. C. (1984). The distribution and transformations of iodine in the environment. Environ. Int. 10, 321–339. 10.1016/0160-4120(84)90139-9

[B190] World Health Organization (2007). Assessment of the Iodine Deficiency Disorders and Monitoring their Elimination. A Guide for Programme Managers. Geneva.

[B191] Xia Y. Gao Q.-M. Yu K. Lapchyk L. Navarre D. Hildebrand D.. (2009). An intact cuticle in distal tissues is essential for the induction of systemic acquired resistance in plants. Cell Host Microbe 5, 151–165. 10.1016/j.chom.2009.01.001 19218086

[B192] Xu F. (1996). Catalysis of novel enzymatic iodide oxidation by fungal laccase. Appl. Biochem. Biotechnol. 59, 221–230. 10.1007/BF02783566

[B193] Yamaguchi N. Nakano M. Takamatsu R. Tanida H. (2010). Inorganic iodine incorporation into soil organic matter: evidence from iodine K-edge X-ray absorption near-edge structure. J. Environ. Radioact. 101, 451–457. 10.1016/j.jenvrad.2008.06.003 18640749

[B194] Yoshida S. Muramatsu Y. Uchida S. (1992). Studies on the sorption of I- (iodide) and IO3- (iodate) onto Andosols. Water Air Soil Pollut. 63, 321–329. 10.1007/BF00475499

[B195] Yu Z. Warner J. A. Dahlgren R. A. Casey W. H. (1996). Reactivity of iodide in volcanic soils and noncrystalline soil constituents. Geochim. Cosmochim. Acta 60, 4945–4956. 10.1016/S0016-7037(96)00305-5

[B196] Yuita K. (1994). Overview and dynamics of iodine and bromine in the environment, 2: iodine and bromine toxicity and environmental hazards. JARQ 28, 100–111.

[B197] Zhu Y.-G. Huang Y.-Z. Hu Y. Liu Y.-X. (2003). Iodine uptake by spinach (*Spinacia oleracea* L.) plants grown in solution culture: effects of iodine species and solution concentrations. Environ. Int. 29, 33–37. 10.1016/S0160-4120(02)00129-0 12605934

[B198] Zia M. H. Watts M. J. Gardner A. Chenery S. R. (2015). Iodine status of soils, grain crops, and irrigation waters in Pakistan. Environ. Earth Sci. 73, 7995–8008. 10.1007/s12665-014-3952-8

[B199] Zifarelli G. Pusch M. (2010). CLC transport proteins in plants. FEBS Lett. 584, 2122–2127. 10.1016/j.febslet.2009.12.042 20036660

[B200] Zimmermann M. B. (2009). Iodine deficiency in pregnancy and the effects of maternal iodine supplementation on the offspring: a review. Am. J. Clin. Nutr. 89, 668S–672S. 10.3945/ajcn.2008.26811c 19088150

[B201] Zimmermann M. B. Jooste P. L. Pandav C. S. (2008). Iodine-deficiency disorders. Lancet 372, 1251–1262. 10.1016/S0140-6736(08)61005-3 18676011

